# The *Pinus taeda *genome is characterized by diverse and highly diverged repetitive sequences

**DOI:** 10.1186/1471-2164-11-420

**Published:** 2010-07-07

**Authors:** Allen Kovach, Jill L Wegrzyn, Genis Parra, Carson Holt, George E Bruening, Carol A Loopstra, James Hartigan, Mark Yandell, Charles H Langley, Ian Korf, David B Neale

**Affiliations:** 1 Section of Evolution and Ecology, University of California, Davis, CA 95616, USA; 2 Department of Plant Sciences, University of California, Davis, CA 95616, USA; 3 Genome Center, Division of Biological Sciences, University of California, Davis, CA 95616, USA; 4 Eccles Institute of Human Genetics, University of Utah, Salt Lake City, Utah 84112, USA; 5 Department of Plant Pathology, University of California, Davis, CA 95616, USA; 6 Department of Ecological Science and Management, Texas A&M University, College Station, TX 77843, USA; 7 Beckman Coulter Genomics (formerly Agencourt Biosciences), Danvers, MA 01923, USA; 8 Institute of Forest Genetics, USDA Forest Service, Davis, CA, 95616, USA

## Abstract

**Background:**

In today's age of genomic discovery, no attempt has been made to comprehensively sequence a gymnosperm genome. The largest genus in the coniferous family Pinaceae is *Pinus*, whose 110-120 species have extremely large genomes (c. 20-40 Gb, 2N = 24). The size and complexity of these genomes have prompted much speculation as to the feasibility of completing a conifer genome sequence. Conifer genomes are reputed to be highly repetitive, but there is little information available on the nature and identity of repetitive units in gymnosperms. The pines have extensive genetic resources, with approximately 329000 ESTs from eleven species and genetic maps in eight species, including a dense genetic map of the twelve linkage groups in *Pinus taeda*.

**Results:**

We present here the Sanger sequence and annotation of ten *P. taeda *BAC clones and Genome Analyzer II whole genome shotgun (WGS) sequences representing 7.5% of the genome. Computational annotation of ten BACs predicts three putative protein-coding genes and at least fifteen likely pseudogenes in nearly one megabase of sequence. We found three conifer-specific LTR retroelements in the BACs, and tentatively identified at least 15 others based on evidence from the distantly related angiosperms. Alignment of WGS sequences to the BACs indicates that 80% of BAC sequences have similar copies (≥ 75% nucleotide identity) elsewhere in the genome, but only 23% have identical copies (99% identity). The three most common repetitive elements in the genome were identified and, when combined, represent less than 5% of the genome.

**Conclusions:**

This study indicates that the majority of repeats in the *P. taeda *genome are 'novel' and will therefore require additional BAC or genomic sequencing for accurate characterization. The pine genome contains a very large number of diverged and probably defunct repetitive elements. This study also provides new evidence that sequencing a pine genome using a WGS approach is a feasible goal.

## Background

Ten years after the first plant genome sequence was completed [1], dozens more have been sequenced but to date no effort has been made to sequence the genome of a gymnosperm species. With 110-120 species spread throughout the Northern Hemisphere, the pines (*Pinus*) comprise the largest genus in the coniferous family Pinaceae of the gymnosperms. Known for their longevity and important ecological roles, pines have also undergone 200-300 million years of evolution separate from their distant angiosperm relatives [2]. A pine genome reference sequence would fill a great evolutionary gap, but it has long been questioned whether such an endeavor was even feasible.

Pine genomes are extremely large (c. 20-40 Gb) [3-6]. These genomes, however, show no evidence of recent polyploidy or chromosome duplication [7-9]. Pine chromosomes (2N = 24) are uniform in both number (2N = 24) and appearance, for they lack major distinguishing physical features [10]. They are also so large and dense that standard karyotyping probes such as rDNA, GC-rich and telomeric repeat sequences failed to produce differential banding patterns among the chromosomes and have complicated karyotyping efforts [11-18]. Pine chromosomes have Arabidopsis-type (*A*-type) telomere repeat sequences (TRS) at their terminal telomeres, as well as substantial centromeric and interstitial sites [19]. A reference karyotype and cytogenetic map was recently produced for *Pinus taeda *L. with improved chromosome spreading techniques and staining probes for two types of rDNA, the *A*-type TRS and centromeric AT-rich regions [20]. A leading commercial timber species native to the southeast United States, *P. taeda *is among the best-characterized pine genomes. There are currently a total of 328628 *P. taeda *expressed sequence tags (ESTs) in NCBI databases, the results of at least five major sequencing projects in root, needle, lignifying and embryonic tissues under varying conditions. These EST sequences were subsequently clustered into 18921 *P. taeda *Unigenes [21]. The current genetic map includes 373 markers across twelve linkage groups [22]. Thus, *P. taeda *is ideal for additional genomic exploration among pines, conifers and gymnosperms.

Several studies report on the complexity of the pine genome. A reassociation study estimated that the *Pinus strobus *L. genome contained 22-26% single-copy elements [23]. When the reassociation calculations were performed with a more accurate genome size estimate, single-copy sequences were estimated to occupy 14% of the genome, or 3100 Mb [3]. This very large single-copy fraction could be due to the presence of large complex gene families in pines, as was evidenced by southern hybridizations performed on *P. taeda *using single-to low-copy gene probes from angiosperms [24,25]. Additionally, the single-copy fraction of the pine genome is enriched for repeats, as it was later shown that at least one fifth of low-copy sequences in *P. taeda *are retroelements and one third contain microsatellite repeats [26,27].

Genomic exploration in conifers has not been limited to pines. *Picea *(spruces) is the most closely related genus to *Pinus *and contains 30-40 species with genome sizes similar to pines. Recent assembly and analysis of four bacterial artificial chromosome (BAC) sequences in *Picea glauca *(Moench) Voss revealed that only one targeted gene was present in each BAC assembly, despite averaging nearly 150 Kb in length [28,29]. Assessment of the surrounding noncoding regions for similarity to repeat database elements revealed that high-complexity repeats comprise 22% and 18% of the two BAC assemblies, where authors noted a prevalence of retroelement-based elements in the results [28]. Repetitive content is thought to be similar in spruces and pines, but there are currently no comparable BAC resources for pine. While a BAC library has been reported for *Pinus pinaster *Ait., no BAC-length sequences have been published for that species [30].

Presented here is the first large-scale sequence survey of a pine genome. This study produced the annotated sequences of ten *P. taeda *BACs using standard Sanger sequencing and assembly methods, as well as 1.66 gigabases of the genome in whole genome shotgun (WGS) sequences from the Genome Analyzer II platform. The linear organization of coding and repetitive elements in ten contiguous genomic sequences is presented visually through computational annotation, similarity to repeat database elements, and several additional innovative repeat analyses. By aligning the WGS reads to the BAC sequences, variation in whole-genome coverage based on alignment stringency is shown. The WGS sequences produced evidence that the three most common repetitive elements in the pine genome together constitute less than 5% of the sequence, and that there appears to be a large number of previously unknown repeat families. While the pine genome is largely repetitive, most of the repeats are highly diverged from one another. Therefore, the main barrier to assembling the nuclear pine genome is not the content of the genome, but the cost associated with its large size.

## Results

### Sequencing and Assembly

**Figure 1:**
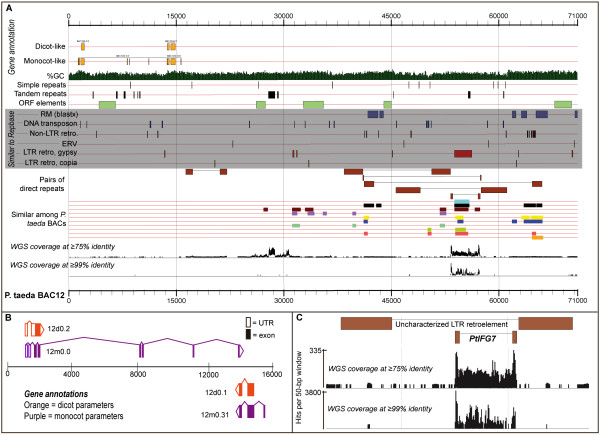
***Pinus taeda *BAC12 (clone Pt314B2) illustrates several new trends found in the pine genome**. (A) The length of BAC12 is shown along the horizontal axis. Shown above the axis are tracks of annotated genes (dicot and monocot parameters), similarity hits to Repbase [RM (blastx); DNA transposons; Non-LTR retroelements; ERV (endogenous retroviruses); LTR retroelements, copia; LTR retroelements, gypsy], and other elements identified in this study (simple repeats, tandem repeats, ORF elements, pairs of direct repeats, and regions of similarity among BACs). The bottom two tracks indicate WGS coverage at ≥ 75% identity and at ≥ 99% identity (B) Genes were annotated with both dicot and monocot parameters. The annotations generally differed in gene structure. (C) Coverage is similar between coverage tracks for active and relatively abundant retroelements in the pine genome such as this nested *PtIFG7*.

Ten *P. taeda *BAC clones were sequenced to an average depth of 10× coverage and assembled into contigs (Table 1). For ease of presentation, each BAC clone will be referenced by its lab designation, given in the leftmost column of Table 1. Among the sequenced clones, coverage ranged from 6× (for the longest clone, BAC3) to over 16× (BAC19). Nine assemblies were resolved into one scaffold that contains linkers and spacers as necessary, while BAC31 assembled into two unoriented contigs. The BAC sequences were deposited in Genbank (Accession nos. GU477256-GU477266). Three sets of whole genome shotgun sequencing were performed with read lengths of 40, 42, and 60 bp. These sets of WGS reads were deposited in the Short Read Archive and assigned accessions SRX017253, SRX017254 and SRX017255, respectively. The GC content of the BACs is similar to the shotgun reads, and the GC content of the *P. taeda *genome falls within typical ranges of angiosperm species (Table 1). The first BAC sequenced, BAC12, is visualized in Figure 1. All ten BACs, their predicted genes and all repeat analyses are shown in Additional file 1, Figure S1 or at http://dendrome.ucdavis.edu/treegenes/gbrowse.

**Table 1:** Summary of *P. taeda *BAC assemblies and whole genome shotgun sequences obtained in this study.

BAC (Clone)	No. contigs (No. final)	Total length (bp)*	Coverage**	%A	%C	%G	%T
BAC3 (Pt285I20)	9 (1)	142351	6.04× BAC	0.291	0.204	0.195	0.311
BAC12 (Pt314B2)	1 (1)	70964	11.57× BAC	0.322	0.186	0.185	0.307
BAC15 (Pt318P9)	1 (1)	67736	14.38× BAC	0.318	0.182	0.190	0.310
BAC17 (Pt321I16)	3 (1)	88546	8.16× BAC	0.303	0.187	0.188	0.323
BAC19 (Pt331B23)	3 (1)	68919	16.12× BAC	0.315	0.178	0.192	0.315
BAC20 (Pt293K22)	4 (1)	61768	15.78× BAC	0.377	0.185	0.188	0.289
BAC21 (Pt348K5)	3 (1)	93889	8.95× BAC	0.310	0.189	0.190	0.311
BAC31 (Pt737O1)	2 (2)	95786	9.31× BAC	0.319	0.179	0.183	0.318
BAC37 (Pt930E21)	6 (1)	128689	6.68× BAC	0.312	0.193	0.189	0.306
BAC40 (Pt921B18)	4 (1)	104081	10.20× BAC	0.301	0.202	0.183	0.313

***40-bp WGS reads***	***-***	***3.28E08***	***0.015× genome***	***0.304***	***0.208***	***0.202***	***0.287***
***42-bp WGS reads***	***-***	***5.38E08***	***0.024× genome***	***0.309***	***0.194***	***0.196***	***0.302***
***60-bp WGS reads***	***-***	***7.98E08***	***0.036× genome***	***0.301***	***0.204***	***0.201***	***0.293***

*Final length of BAC assembly after vector sequence was removed and linked contigs were joined with N blocks.

**BAC coverage was calculated by dividing the total number of P20 bases by the total amount of pine sequence in each scaffold assembly. Genomic coverage of WGS reads was determined by dividing the total base pairs by the genome size, 2.2E10 bp.

### Gene Content

The BAC library was probed for sequences similar to ten *P. taeda *genes known to be involved in the phenylpropanoid pathway and lignin biosynthesis (see Methods for probes). The probes were derived from EST contigs amplified from genomic DNA and either differ significantly from the published mRNA or lack corresponding mRNA sequence altogether. The BACs contained thirteen regions with similarity to six probes; *cinnamyl-alcohol dehydrogenase (cad), caffeoyl-CoA O-methyltransferase (ccoaomt), caffeate O-methyltransferase (comt)*, *LIM transcription factor 1(ptlim1), S-adenosyl methionine synthetase 1 (sam1) and S-adenosyl methionine synthetase 2 (sam2) *(Additional file 2, Table S1). The regions of similarity with these genes averaged 211 bp with 87% nucleotide identity. Four probes did not significantly match any BAC sequence. Based on 100% nucleotide identity with its probe, it appears that a novel *ccoaomt *may have been captured in BAC19. The other nine BACs contained regions with partial similarity to the probes. In order to determine the coding potential of these regions and to discover untargeted genes in the BACs, the MAKER automated annotation software was run with both dicot and monocot parameters (Table 2) [31]. The runs indicate that approximately 1% of the sequence in these BAC clones may be protein-coding. Eight of the ten BACs contain predicted genes, while BAC3 and BAC37 contain no predicted genes. The two runs produced similar results, with dicot and monocot genes occupying the same or overlapping regions, but exon-intron structure generally differed between the two runs (Figure 1B; Additional file 3, Table S2). On average, the dicot-like genes occupy less genomic space but produce longer mRNAs and peptides than the monocot-derived genes. The dicot-like genes contained an average of 2.9 exons (average 219 bp) and the monocot-like genes showed an average of 2.8 exons (average 187 bp). Both runs predicted four small introns per gene, with average lengths of 350 and 842 bp, respectively. The dicot run predicted a total of 18 genes, two of which lack consensus start or stop codons (Additional file 2, Table S1). The monocot run also predicted 18 genes but seven genes lack consensus start or stop codons, making them gene fragments. All gene predictions employ consensus splice signals at exon-intron junctions. Fourteen of the 18 total predicted genes were annotated against protein or nucleotide genes sequences from organisms other than *P. taeda*. The results of the MAKER runs, including gene annotations and supporting evidence, can be viewed in Additional file1, Figure S1.

**Table 2:** Summary of elements in ten annotated pine BACs, as identified by MAKER (white background) and through additional repeat analyses performed in this study (shaded background).

	BAC3	BAC12	BAC15	BAC17	BAC19	BAC20	BAC21	BAC31	BAC37	BAC40	ALL
No. dicot-like genes	0	2	2	2	1	1	2	1	0	7	18
Dicot-like gene content	0	3.0%	4.7%	4.5%	3.7%	2.5%	2.8%	1.5%	-	6.5%	2.6%
No. monocot-like genes	0	2	2	1	1	1	2	1	0	8	18
Monocot-like genes content	0	20%	3.9%	3.7%	11.3%	2.5%	1.9%	1.5%	-	5.8%	4.2%

TRANSPOSONS	72	46	31	73	47	51	64	79	81	55	599
DNA transposons	23	11	11	19	19	15	28	22	24	18	190
ERVs	4	2	2	6	1	1	2	3	0	6	27
Non-LTR retroelement	7	13	6	18	12	16	7	28	18	7	132
LTR retrotransposons	38	20	12	30	15	19	27	26	39	24	250
*Gypsy*-like	26	7	9	17	6	14	15	13	26	10	143
Named elements*	***4***	***1***	***2***	***1***	***1***	***1***	***1***	***1***	***1***	***1***	*14*
*Copia*-like	17	3	3	13	6	4	12	10	11	13	92
Named elements*	***1***	***0***	***1***	***2***	***1***	***1***	***0***	***2***	***2***	***0***	*10*

INTEGRATED VIRUSES	0	0	1	0	0	0	0	1	0	1	3
OTHER REPBASE	0	0	0	1	0	2	2	1	1	1	8
SIMPLE REPEATS	16	10	4	9	12	2	22	18	41	18	152

TOTAL NO. REPBASE HITS	88	56	36	83	59	55	88	99	123	75	762
***Similar to Repbase or RM***	***18%***	***12%***	***12%***	***15%***	***17%***	***19%***	***12%***	***17%***	***15%***	***9%***	***17%***

Tandem repeats/minisats**	13	11	10	14	23	14	22	45	21	41	214
Direct rpts/potential LTRs**	40	12	10	10	4	6	12	24	27	16	161
Putative ORF elements**	11	5	3	8	5	6	8	3	14	7	70

NO. ADD'L REP. ELEMENTS	64	28	23	32	32	26	42	72	62	64	445
***New Repetitive Content***	***72%***	***54%***	***50%***	***59%***	***34%***	***75%***	***44%***	***93%***	***59%***	***38%***	*63%*

Repetitive content*** at 75% threshold (similarity)	***81%***	***83%***	***80%***	***82%***	***70%***	***86%***	***76%***	***85%***	***75%***	***82%***	***80%***
Repetitive content*** at 99% threshold (identity)	***25%***	***21%***	***22%***	***24%***	***15%***	***35%***	***19%***	***30%***	***15%***	***29%***	***24%***

*The occurrence of novel gypsy-like and copia-like elements (underlined) was manually examined as described in the text.

**See Methods for a description of the discovery of putative ORF elements, tandem repeats and direct repeats.

***The percentage of sites in each BAC assembly that aligned with one or more WGS reads at thresholds of 75% and 99% identity.

Of the 18 predicted genes, twelve have similarities to Interpro domains [32] with an E-value < 1e-05 (Additional file 4, Table S3). The putative *ccoaomt *gene on BAC19 is only one of several genes that, if they function as predicted, could be homologues to genes involved in lignin biosynthesis. Two additional *ccoaomt *genes (BAC20, BAC31), three *comt *genes (BAC12, BAC17, BAC40), a LIM transcription factor (BAC15), and one *sam1 *(BAC21) are predicted in regions of similarity to their probes (Additional file 1, Figure S1). The set of predicted genes also includes untargeted genes such as a member of the *4cl *family (BAC12), a glycosyl transferase gene (BAC15), a *SMARCA3 *helicase (BAC21), three kinases (BAC40) and a reverse transcriptase that likely belongs to a transposon (BAC17). Three predicted genes do not show any similarity to Interpro (BAC40). Analysis of upstream promoter regions shows that while most of these twelve genes do contain promoter elements, many of the predicted genes lack appropriately located TATA or CAAT boxes that are generally required for transcription (Additional file 5, Table S4). Based on the presence of consensus start and stop codons, significant Interpro hits, and > 97% nucleotide identity with *P. taeda *ESTs, only three methyltransferases (*comt *in BAC12, *ccoaomt *in BAC19 and *ccoaomt *on BAC20) may be novel protein-coding genes. The other 15 predicted genes are most likely inactive pseudogenes.

### Repeat Content

As can be seen in Additional Figure 1, the noncoding component of the ten pine BACs is composed of highly diverse repetitive elements. The MAKER output reports that BACs showed similarity to nearly 600 complex Repbase elements in less than one megabase of pine genomic sequence [33] (Table 2; Additional file 1, Figure S1). These include simple sequence repeats such as GC-and AT-rich areas and complex repeats such as LTR and non-LTR retrotransposons, DNA transposons (including hAT, MuDR and Helitrons), endogenous retroviruses (ERV), and other repetitive elements as defined by Repbase. The sources of the accessions with similarity to the pine BACs vary widely, including five gymnosperm species, 15 dicot and eight monocot species, 11 animals, three fungal species, a moss and one multicellular alga. Only 4% of all hits to complex Repbase repeats are to gymnosperm-derived repetitive elements, reflecting the relative lack of genomic resources for this clade.

#### Conifer-specific LTR retroelements

Similarity to gymnosperm accessions was used to identify conifer-specific LTR retroelements in the BACs. The *IFG7 *gypsy-like retroelement in *Pinus radiata *D. Don [Genbank: AJ004945] and *PpRT1 *in *P. pinaster *Aiton [Genbank: DQ394069] are known to be pervasive in the pine family [34,35], and four *P. taeda *BACs show strong similarity (80-93% identity over an average 3900 bp) to *IFG7 *(Figure 1C). These four *IFG7*-like sequences are over 90% similar to each other, so they represent four partial copies of a novel *P. taeda *equivalent to *IFG7 *that is designated *PtIFG7 *(Table 2; Additional file 1, Figure S1). The high level of sequence conservation suggests that the retroelement may be relatively young and still active. The fact that *PtIFG7 *is actively transcribed is further supported by the fact that WGS coverage of the *PtIFG7 *elements is similar at 75% and 99% identity (Figure 1B) and, ultimately, that the sequence shows 100% nucleotide identity with at least seven *P. taeda *ESTs. Note that while *PtIFG7 *is present in four BACs, they are each interrupted by other elements or truncated by the end of the BAC assembly (Table 2; Additional file 1, Figure S1).

In BAC21, a nearly full-length match to *PtIFG7 *shows only 66-73% nucleotide identity. The region is flanked by 94% similar direct repeats and also contains 89 bp similar to the *Gypsy8 *element in *Vitis vinifera *L. Neither the LTRs nor Vv*Gypsy8 *are similar to *PtIFG7*. This novel relative of *PtIFG7 *is tentatively described as *PtIFG7-2*. As can be seen in Additional file 1, Figure S1, the LTRs of the putative *PtIFG7-2 *in BAC21 are tightly flanked by large (1100-1750 bp) similarity hits to copia-like elements from *Medicago truncatula *Gaertn., *Populus trichocarpa *Torr. & Gray and *Oryza sativa *L., so the *PtIFG7-2 *appears to be nested inside a larger uncharacterized copia-like element. The *PtIFG7-2 *element shows 100% nucleotide identity to a single EST, so it is possible that *PtIFG7-2 *is also actively transcribed in the genome. A second relative of the *PtIFG7 *element can be found in BAC37. Instead of one long similarity hit, however, this new *PtIFG7-3 *element is fragmented into five pieces and contains a number of unrelated repetitive elements and similarity hits. The pieces of the *PtIFG7-3 *element average 66% nucleotide similarity to *PtIFG7 *and 75% similarity to *PtIFG7-2*. Since the similarity seen here is below 80%, we consider *PtIFG7-3 *a distinct but related element to *PtIFG7 *and *PtIFG7-2*, although we note that classification of these elements into classes and families is confused by the level of divergence among them. One similarity hit each to LTR retroelements in *Pinus elliottii *Engelm. and *P. thunbergii *Parl. average only 230 bp in length and were uninformative.

Two additional novel LTR retroelements were identified based on similarity to *Picea *elements (Additional file 1, Figure S1). BAC37 contains a 2300-bp region showing 84-87% identity with the *PGGYPSYX1 *(*Spcl*) retroelement in *Picea glauca *[Genbank: AF229252] [36]. The region is flanked by 90% identical LTRs, so this new gypsy-like element is tentatively described as *PtGypsyX1*. The *PtGypsyX1 *retroelement is also present twice in BAC3, and the three copies described here are 87-89% similar at the nucleotide level. A strong hit in BAC3 to the *PGCOPIAX1 *(*Spdl*) element from *Picea glauca *[Genbank: AF229251] is situated between direct repeats located about 10 Kb apart, a space shared with hits to copia-like elements in *M. truncatula *and *V. vinifera*. It remains unclear whether this LTR-flanked portion of BAC3 represents a single intact element, but the portion showing 79% nucleotide identity with the spruce element is tentatively identified as a fragment of the newly described *PtCopiaX1 *element. The *PtGypsyX1 *and *PtCopiaX1 *have different WGS coverage profiles between the thresholds (deep coverage at 75% identity, low coverage at 99%), suggesting that they are older than the *PtIFG7 *family. However, the *PtGypsyX1 *near position 60 Kb in BAC3 shows 100% nucleotide identity with six full-length *P. taeda *ESTs, so this element appears to be actively transcribing and proliferating in the genome. The *PtCopiaX1 *element in BAC3 has a relatively high WGS coverage in the LTRs and the portion corresponding to the assembled *TPE1*-containing element, but an internal portion (128-134 Kb) has a distinctly lower coverage than the *PtCopiaX1 *in which it is nested. This copy of the *PtCopiaX1 *element does not show similarity to any *P. taeda *ESTs, but the unrelated internal region (which happens to correspond with a putative ORF element) shows 100% nucleotide identity with two *P. taeda *ESTs. This particular copy of *PtCopiaX1 *is dead, and in this case appears to contain an active transcribed repetitive element of unknown origin.

#### Angiosperm-derived LTR retroelements

Sequences similar to Repbase or RepeatMasker database repeats contribute 23% to the total BAC assemblies, and contributions among BACs range from 19% in BAC40 to 33% in BAC31 (Table 2). If 1% of the BACs are considered coding (Table 2; Additional file 3, Table S2), this leaves approximately 75% of the BAC sequence uncategorized. To determine if the uncategorized sequence was single-copy or repetitive, WGS reads were aligned to the BACs and the alignment depth at each position was observed. The total coverage represented by the WGS was 0.036x of the genome, so most single-copy regions should appear unaligned. Repetitive regions, however, are expected to have multiple aligned reads. Alignments were categorized as either ≥ 99% identical or ≥ 75% identical. At the 99% threshold, most (77%) of the BAC sequence can be considered single-copy. There are a few regions, such as the newly characterized *PtIFG7*, which are repetitive at this stringent threshold (Figure 1C). At the 75% threshold, most (80%) of the BAC sequence is repetitive. Based on WGS coverage of the *PtGypsyX1 *elements in BAC3 and BAC37, it is estimated that the genome contains 65000 to 72000 copies with ≥ 75% similarity and 600 to 2000 copies with ≥ 99% similarity to the copies in the BACs. Using the *PtCopiaX1 *in BAC37, the copy number of this element is estimated to be around 84000 at the 75% threshold and just over 1000 at the 99% threshold. There also appear to be several new families of repeats as indicated by similarity to non-gymnosperms, with coverage profiles that appear to confirm and delineate repetitive units (indicated by red or green boxes in Additional file 1, Figure S1).

The average length of similarity between pine BAC sequence and non-gymnosperm Repbase accessions is 155 bp, far shorter than full-length transposons, and most hits were not suggestive of full-length repetitive elements in pine. The MAKER output also included 62 significant blastx hits to RepeatMasker coding sequences, averaging 753 bp of coding similarity to reverse transcriptases or polyproteins in angiosperm retroelements. Complicating novel retroelement identification is the fact that a single gymnosperm element often shows similarity to numerous angiosperm repeat accessions. Using the Repbase and RepeatMasker similarity hits coupled with the WGS coverage profiles, 15 novel partial or full-length pine LTR retroelement sequences were tentatively identified based on similarity to non-gymnosperm accessions (Additional file 1, Figure S1). Seven of these are gypsy-like elements and eight appear to be copia-like LTR retrotransposons. Informative Repbase accessions originate from *Glycine max *(L.) Merr., *Populus trichocarpa*, *Oryza sativa, Vitis vinifera*, *Zea mays *L.*, Cicer arietinum *L. *Lotus corniculatus *L., and the novel pine retroelement sequences are indicated in Additional file 1, Figure S1. The 15 novel angiosperm-derived LTR retroelements tentatively identified in this study are also older and less frequent in the genome than *PtIFG7*, as evidenced by lower WGS coverage profiles and the presence of unrelated repetitive elements between the LTRs.

#### Direct, tandem and simple repeats

At least 161 direct repeats were found among the BACs, with individual BACs ranging from four pairs (BAC19) to 40 pairs (BAC3). As discussed above and illustrated in Additional file 1, Figure S1, about 30 pairs of direct repeats belong to identifiable LTR retroelements, and this is almost certainly an underestimate. Many of the direct repeats appear to simply be repeated sequences. As a case in point, the *PtIFG7 *element in BAC12 contains a 142-bp sequence that is also found about 16 Kb upstream of the *PtIFG7 *element (Figure 1). This small repeated sequence does not show similarity to any known sequence, and it is unclear how one copy was inserted into the *PtIFG7 *retroelement. The average nucleotide identity between direct repeats was 86% and ranged from 53% to 99%.

Tandem repeats, or minisatellites, have not to date been investigated in a gymnosperm genome. Tandem Repeat Finder identified 214 repeats of units 5-200 bp in length in the ten *P. taeda *BACs (Figure 1; Additional file 1, Figure S1) [37]. BAC12 shows a large defined peak in WGS coverage at the 50-bp tandem near the middle of the BAC (Figure 1). The common occurrence of tandem repeats in the BACs and the peak in WGS coverage profiles of the BAC12 tandem together offer evidence that tandem repeats may be dispersed throughout the genome and contribute to genome complexity. Simple repeats are difficult to quantify based on WGS coverage, but over 150 were identified among the ten *P. taeda *BACs (Table 2). Previous studies have shown that simple repeats and microsatellites are found throughout the pine genome [27,38,39]. Using the unmasked MAKER runs, there were also 70 putative ORF elements identified (Table 2). These are consistently predicted reading frames that are not near any putative protein-coding genes or pseudogenes in the BACs. As can be seen in Figure 1 and Additional file 1, Figure S1, the putative ORF elements typically fall within repetitive regions of the BACs as indicated by WGS coverage. They are not confirmed to belong to, or consist of, novel repetitive elements, but the suggestions are offered here as researchers begin exploring the evolutionary history of the conifer genome and identify more novel gymnosperm-specific families of repeats.

*Genome-wide consensus repeats*. To derive the full-length sequence of the most common repetitive elements in the genome, a highly permissive assembler was used to build the repeats from WGS reads (Table 3). Since no two reads are expected to truly overlap, the assembled elements are consensus sequences containing the most common base at each position. The most abundant element is 3896 bp long (with one LTR) and is 90% identical to the 1663-bp *TPE1 *[Genbank: Z50750], an internal copia-like sequence used to assay the occurrence of such retroelements in a variety of large gymnosperm genomes [40]. Alignments to WGS reads show that this element comprises approximately 1.6% of the *P. taeda *genome. The assembled *TPE1*-containing element is present in BAC3 in four fragments (> 90% identity to consensus) within the *PtCopiaX1 *element located there (Additional file 1, Figure S1). Although the *PtCopiaX1 *BAC3 was determined to be interrupted and inactive, the *TPE1*-containing consensus shows > 97% full-length nucleotide identity with at least five *P. taeda *ESTs. The second most common element in the genome is *PtIFG7*, of a similar size and abundance (3686 bp, 1.3% of the genome) to the *TPE1*-containing element. The third repeat assembled was a 50-nucleotide tandem repeat (cen-rpt), comprising 0.27% of the genome. This may correspond to the centromere repeat, and if so, represents the first example from a gymnosperm. The sequences of the three assembled elements are presented in Additional file 6, Table S5. A separate assay of the WGS reads found that 0.24% contains the A-type telomeric repeat (tel-rpt: TTTAGGG). This suggests that this telomeric repeat accounts for roughly 50-55 megabases of the genome.

**Table 3:** Three common repeats were assembled from a pool of 21 million WGS reads representing 3.9% of the *P. taeda *genome.

	No. reads	*TPE1/copia*	*PtIFG7*	cen-rpt	tel-rpt*
**No. WGS reads**	2100000	330219	281712	57524	50494
**Est. genome portion**	3.5%	1.57%	1.34%	0.27%	0.24%
**Total base pairs**	87,000,000	350000000	300000000	60000000	53000000
**Est. element length**	--	4200	4000	50	7
**Est. copies in genome**	--	82000	74000	--	--
**Ave. no./chromosome**	--	6900	6100	--	--
**Ave. bp/chromosome**	36000000	29200000	25000000	2500000	22000000

*Also reported are the results of a separate assay of the WGS reads for similarity to the consensus plant telomeric tandem repeat (tel-rpt; TTTAGGG).

## Discussion

The purpose of this study was to explore the content and organization of a conifer genome and assess the feasibility of sequencing and assembling a reference pine genome. In the course of the study, several interesting aspects of the pine genome have emerged. Analyses of the structure and content of ten P. taeda BACs suggest that pseudogenes may be common in the pine genome and that isolated repetitive elements such as LTR retrotransposons can be discerned from a background of fragmented fossil repeats of unknown origin. The frequency of partially conserved coding regions in the genome is consistent with the numerous hybridizations observed to probes for single-copy genes [25]. In these ten BACs, apparent pseudogenes appear to occur five times more frequently than true potentially functional protein-coding genes, but the BACs were enriched for coding sequences and represent only 0.0042% of the genome. Whether this ratio extends to the entire genome remains to be seen. The common occurrence of pseudogenes in these BACs is consistent with the two conclusions from other studies: (1) most pine genes have many paralogues or pseudogenes and (2) pine genes are relatively compact. The accuracy of these and any other computational gene predictions in pine are limited by our incomplete knowledge of codon or promoter usage in pines. Gene identification in this complex genome must be achieved through deep transcriptome or genome sequencing, as well as experimental validation of expression. It is, however, critical to note the pine genes do appear to be quite compact relative to the dauntingly vast genome size (Additional file 3, Table S2). Thus, intermediate sequencing strategies that can leverage a deep transcriptome may efficiently assemble the primary functional genomic domains. Genetic localization of such gene islands can serve both as an experimentally valuable (if sparse) scaffold and a solid foundation for completion of a reference genome sequence.

Previous studies showed that the pine genome is highly repetitive, but not predominantly high C_0_T [41]. The current study indicates that the reason for this is that there are many diverged repeats, and no single conserved repetitive element constitutes more than 2% of the genome. Depending on how one conducts a hybridization experiment, one could see extremely different results. Under stringent conditions (99% identical), only 24% of the genome is repetitive. Under more permissive conditions (75% identical), 80% of the genome is repetitive. The present sequence survey of the pine genome supports the findings of hybridization studies by suggesting a massive 'low-copy' fraction and a very small 'high-copy' fraction containing a few repeat families that occur fewer than 100000 times in the 22-Gb genome. At this point, little else is known about LTR retroelement families and other types of repeats in pine except that they appear to be numerous and highly diverged. The *IFG7 *family of elements is currently thought to exist only in *Pinus*, while *PtCopiaX1 *looks to be shared with *Picea*. While it is clear that a significant amount of work remains in order to truly determine the age of any LTR retroelements in pine, the fact that *PtCopiaX1 *is about 85% identical with *PGCOPIAX1 *suggests that this element may have been present in the common ancestor of the two genera. Considering that *Pinus *and *Picea *diverged approximately 140 million years ago [42], this copia-like element could be ancient. Alternatively, the retroviral progenitor of *PtGypsyX1 *and *PGGYPSYX1 *may have inserted multiple times during the evolution of conifers. In either case, two aspects of the LTR retroelements described in the BACs are fundamentally different from those found in other plant genomes: age and degree of divergence.

Analyses of LTR retrotransposons have been performed in a wide variety of plant genomes including eudicot, monocot and gymnosperm species. The abundance and organization of these repetitive elements are loosely correlated with clade: eudicots generally have fewer and smaller repetitive elements than other clades, while the monocots are known for their high LTR retroelement activity and rapidly changing genomes. Now, conifer genomes can be distinguished from angiosperm genomes by the old age and high degree of divergence in both their intact and fragmented LTR retrotransposons compared to the younger LTR retroelements in angiosperm genomes. A rapid increase of retroelement density in *Oryza sativa *occurred around eight million years ago, but unequal homologous recombination subsequently removed two-thirds of that LTR retroelement sequence and left a genome consisting of only 26% retrotransposons [43]. The *Sorghum bicolor *L. (Moench) genome, twice the size of *Oryza sativa*, is composed of 55% retrotransposons that have mostly inserted in the last two million years [44]. The *Zea mays *genome, three times as large as *Sorghum bicolor*, contains roughly 80% retroelements, most of which are not present in the orthologous *Sorghum bicolor *[45]. This implies that most of the LTR retroelements in the *Zea mays *genome inserted since its divergence from the *Sorghum *genome about 16 million years ago [46]. A survey of two linked *Triticum aestivum *L. BACs identified eleven LTR retroelements, all determined to have inserted less than 10-14 million years ago [47]. The majority of LTR retroelements observed in these studies of grass genomes are relatively young and clearly distinguishable. In contrast, only two conifer-specific LTR retroelements in the BACs (*PtGypsyX1 *and *PtCopiaX1*) may be still active after 140 million years, and the *IFG7 *family of gypsy-like elements appears to be active in both subgenera of *Pinus *[34], which diverged approximately 110 million years ago [2].

The bulk of the pine genome remains an enigma. It appears to contain diverged fragments of an extremely diverse set of repetitive elements. The analyses performed here virtually exhaust similarity-based identification so future repeat discovery will require additional genomic sequence. In any case the implications of the ancient and diverged nature of the repeats that comprise a majority of the pine genome are both theoretical and practical. The conservative karyotype and the lack of rapid turnover of the vast and repetitive portion of the pine genome raises questions about the possible functional roles leading to evolutionary constraint, and about potentially unique mechanisms for the maintenance of genomic integrity. In terms of the challenging goal of a reference pine genome sequence, this predominance of a *highly diverged *repetitive component is critical since large amounts of identical dispersed repeats are inevitable sources of gaps in an assembly.

## Conclusions

In this detailed analysis of ten *Pinus taeda *BACs, we identified three putative protein-coding genes and at least fifteen pseudogenes or gene fragments. Examining the BACs in the context of 34.3 million WGS reads and 600 similarity hits to repeat databases, we found that 9.1% of the BACs had high WGS coverage or significant similarity to one of three positively identifiable conifer-specific LTR retroelements (*PtIFG7*, *PtGypsyX1 *and *PtCopiaX1*). An additional 12% of the BACs contain relicts of LTR retrotransposons that were tentatively identified as copia-like or gypsy-like based on similarity to angiosperm repeats. Simple repeats and imperfect tandem repeats together represent less than 4% of the total BAC sequence. Direct repeats larger than 100 bp occupy nearly 30% of the BAC sequence, but two thirds of pairs are less than 90% similar (none are identical) and only a minority are clearly associated with a retroelement. The majority of the BAC assemblies were comprised of ancient repetitive sequences. This is in sharp contrast to the *Oryza sativa*, *Sorghum bicolor *and *Zea mays *genomes, where recently amplified and minimally diverged LTR retrotransposons occupy much of the intergenic space. There is no evidence of recent segmental or block duplications within the BACs.

**Figure 2:**
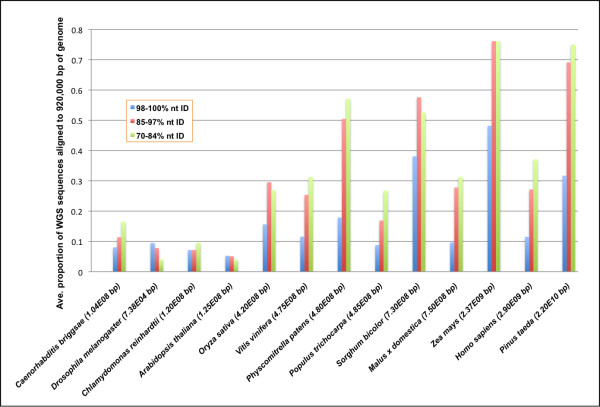
**Comparison of repeat content among twelve sequenced genomes and *Pinus taeda***.

These preliminary insights into the nature of repeats in the pine genome provide compelling evidence that sequencing a large pine genome such as *P. taeda *is certainly within reach. Sequencing a genome this large using a rigorous BAC-by-BAC approach, however, would entail an exorbitant time and monetary cost. Can *P. taeda *be sequenced and assembled from WGS reads? It is difficult to answer this definitively because no one has attempted to assemble a 22-Gb genome to date, but one can gain insight by comparing the repeat content to sequenced genomes. The *P. taeda *genome contains fewer repeats that are nearly identical (98-100%) than either *Sorghum bicolor *or *Zea mays *(Figure 2, see Methods for description of this computational comparison). The *Sorghum bicolor *genome was successfully assembled from WGS Sanger reads, and the *Pinus *genome contains fewer recently amplified, and highly similar, repeats than *Sorghum *[44]. The degree of divergence within the genome should facilitate assembly of a draft sequence for *P. taeda*. Based on the age and diversity of pine repeats, it may even be possible to assemble the genome using a whole genome shotgun strategy based on several platforms and a range of insert sizes. Regardless of strategy, the massive *P. taeda *genome will surely challenge the limits of contemporary sequencing technology.

## Methods

### BAC Clone Sequencing and Assembly

Loblolly pine BAC library Pt_7Ba (Clemson University Genomics Institute [CUGI], Clemson, SC) was screened with multiplexed ^32^P-labeled PCR amplicons from ten genes mapped to quantitative trait loci (QTL) associated with wood chemical traits in loblolly pine [48-50] (Additional file 6, Table S5). Hybridizations identified 256 positive clones, and a random 48-clone subset of the positives was obtained from CUGI. The BAC DNA was isolated with the Colony Fast-Screen| Kit (Epicentre Biotechnologies, Madison, WI) and sized relative to BAC-Tracker| Supercoiled DNA Ladder (Epicentre) using SYBR, Gold (Molecular Probes, Eugene, OR) and agarose gel electrophoresis. Subsequently, ten BACs were selected that showed single BAC insert bands and were different from each other. Glycerol stocks were sent to Beckman Coulter Genomics (Danvers, MA) for subclone library construction and sequencing.

For each BAC, a shotgun library was prepared from a single clone inoculated to 500 mL of LB with 12.5 μg/mL chloramphenicol. High molecular weight DNA was produced using the Qiagen (Valencia, CA) Large-Construct Kit. The DNA was randomly sheared using a Genemachines Hydroshear (Genomic Solutions, Ann Arbor, MI). The sheared DNA was end-repaired with Epicentre End-It| End-Repair Kit and size selected for inserts from 2 to 4 kilobases to produce libraries with average insert sizes of 2 Kb, 3 Kb and 3.5 Kb. The insert DNA was ligated to pUC19 high copy plasmid vector (Fermentas, Glen Burie, MD). The ligations were transformed into DH10B T1r E.coli cells (Invitrogen, Carlsbad, CA) and plated on LB agar with appropriate carbenicillin, X-gal and IPTG concentrations. Transformation mixes were quality controlled via enzyme digest and arrayed into 384-well plates containing LB freezing medium. Subclone DNA templates were sequenced in 384-well format, using BigDye^® ^Version 3.1 reactions on ABI3730xl instruments (Applied Biosystems, Foster City, CA) with the forward and reverse reactions (paired ends) being done in the same plate to maximize the paired end rate. Thermal cycling was performed using 384-well Thermocyclers (Applied Biosystems). Sequencing reactions were purified using Agencourt's CleanSeq^® ^dye-terminator removal kit.

All reads were processed using PHRED base calling software and constantly monitored against quality metrics using the PHRED Q20 [51,52]. The quality scores for each run were monitored through Agencourt's Galaxy LIMS system. A passing read was defined as an average high quality PHRED score of 20 or higher for at least 100 bases. Typical average read-lengths extended 500-600 bp. The Arachne Whole Genome Assembler [53], coupled with Agencourt's LIMS system, was used to assemble the BAC sequences. Assemblies were viewed in CONSED [54,55].

### Computational Annotation of BAC Assemblies

Annotations for the *P. taeda *contigs were prepared using the program MAKER, a genome annotation pipeline that identifies repetitive elements, aligns EST and protein homology evidence, prepares *ab initio *gene predictions, calculates quality control metrics, and synthesizes these data into final genome annotations.

The EST/cDNA sequences used by MAKER were derived from *P. taeda *and were combined with EST/cDNA sequences from all other Pinaceae species found in dbEST [56]. The UniProt/Swiss-Prot [57,58] protein database was used as the protein homology database for the MAKER run. Repeat elements were identified using a MAKER internal transposable element database, the RepBase repeat library in conjunction with RepeatMasker, and pre-computed repeats from the program CENSOR [33] passed to MAKER via the algorithm's GFF3-passthrough option.

The total length of the preliminary contig set (923817 bp) was too short to accurately train the *ab inito *gene predictors specifically for the *P. taeda *genome. Instead a hybrid approach was taken by using existing training parameters from both monocot and dicot plant species to produce gene predictions in separate MAKER runs. Because MAKER uses evidence alignments to produce "hints" which are then sent to the *ab initio *gene prediction algorithms that can accept them, prediction algorithms that run inside the MAKER pipeline are capable of producing improved gene models even when the training parameters are imperfect. After producing a pool of possible *ab initio *and "hint-based" gene predictions, MAKER chooses those that are best supported by EST and protein homology evidence alignments using internal quality control metrics [31,59] and promotes them to the status of genome annotations.

MAKER was first run using the *ab initio *gene prediction algorithms SNAP [60], Augustus [61,62], and GeneMark [63] trained for *Arabidopsis thaliana *and FGENESH [64] trained for a generic dicot species (the exact species was not specified in the FGENESH documentation). The second run of MAKER was performed using SNAP and GeneMark trained for *Oryza sativa *in conjunction with Augustus trained for *Zea mays*. Both sets of MAKER-produced gene models were saved in GFF3 [65] format and simple intron/exon structure statistics were calculated against them using the program Eval [61,66] The MAKER runs were viewed and evaluated using the Apollo Genome Annotation Curation Tool [67]. The peptide sequences corresponding to both sets of MAKER gene predictions were searched for conserved protein domains using Interproscan with default parameters [68] against the Interpro protein signature database.

### High-throughput Whole Genome Shotgun Sequencing

Whole genome shotgun sequencing was performed on diploid DNA from the same individual used to construct the BAC library, using the high-throughput Illumina Genome Analyzer II sequencing platform. Genomic DNA library construction was carried out using the Illumina genomic DNA sample preparation kit according to manufacturer's instructions, except that paired end specific oligonucleotides were used instead of the single read oligonucleotides. Starting material was 80 ul of pine genomic DNA at a concentration of 62.5 ng/ul sonicated in a Diagenode Bioruptor for 15 cycles of 30" on maximum power then 30" rest. Following paired end adapter ligation, fragments of approximate size 400-425 bp were gel purified and PCR amplified using the paired end Illumina library PCR primers (primers 1.0 and 2.0). After AMPure purification (Beckman Coulter Genomics), the sample was applied to an Agilent Bioanalyzer for quantitation. Based on the bioanalyzer-reported sample concentration, the library was applied to a flow cell at 5 pM using v1 cluster reagents. Sequencing was performed on an Illumina Genome Analyzer II using version 2 sequencing reagents for 40, 42 and 60 cycles. Basecalling was carried out using the Illumina GA Pipeline v1.3. The WGS sequencing was carried out at the University of California, Davis, Genome Center.

### Additional Element Characterization in BAC Assemblies

As previously described, the MAKER automated annotation pipeline was customized for both gene prediction and repeat identification in the ten *P. taeda *BAC assemblies. MAKER reported simple sequence repeats, as well as similarity to Repbase accessions and the MAKER internal transposable element database. Since only a handful of complex repetitive elements have been characterized in conifers, it is expected that this similarity-based repeat landscape described by MAKER is incomplete.

Several additional methods were included to complete the identification of putative repetitive elements in the BAC assemblies. Tandem Repeats Finder was used to locate tandemly duplicated units of 5-200 bp, Gepard [69] was used to produce dotplots in order to visualize longer direct and inverse repeats within each BAC, and discontiguous megablast (word size 11, match/mismatch = +1/-1, gap open/extension cost= 2/2) was used within each BAC to delineate direct repeats of minimum length 100 bp that span at least 500 bp of putatively noncoding sequence. The resulting pairs of direct repeats are presented in this paper as potential long terminal repeats of uncharacterized LTR retrotransposons. The results of MAKER run with dicot parameters on unmasked pine BACs were also examined for evidence of nongenic open reading frames (ORFs) that may correspond to 'novel' complex repetitive elements such as DNA transposons or LTR retrotransposons.

Regions were identified where at least two MAKER gene-finding tools predicted ORFs, but the sequence failed to show enough similarity to EST and protein databases to be annotated as protein-coding genes. Each putative nongenic ORF element shows significant similarity to at least one known repetitive element and is described using the longest ORF (minimum length 240 bp) among similar predictions.

### Whole Genome Shotgun Sequence Analysis

Two consensus transposons and a putative centromeric tandem repeat were assembled from a pool of 40 and 42-bp WGS reads using nugtohs.pl (unpublished). In order to assess genome-wide occurrence of putative genic and repetitive elements in the BAC assemblies, 60-bp WGS reads were aligned to each BAC sequence with BLASTN and post-processed with a Perl script. This produced two WGS-coverage maps of each BAC; one coverage map optimized for WGS-to-BAC alignments showing 99% nucleotide identity (score threshold 55) and one map optimized to count alignments at or above 75% nucleotide identity (score threshold 24). The coverage maps are reported in hits per base pair in .sgr formats that were initially analyzed using the Integrated Genome Browser [70]. Genome-wide copy number of BAC elements were computed by averaging hits per base pair along the length of each element and calculating the ratio of this value to the estimated genome coverage (0.036×) provided by the 60-bp reads.

### Assessment of Pine Genome for Sequencing and Assembly

To assess the *P. taeda *genome for sequencing and assembly, the repeat content of the genome was compared to twelve previously sequenced genomes: *Caenorhabditis briggsae *[71], *Drosophila melanogaster *[72], *Chlamydomonas reinhardtii *[73], *Arabidopsis thaliana *[1], *Oryza sativa *[74], *Vitis vinifera *[75], *Physcomitrella patens *[76], *Populus trichocarpa *[77], *Sorghum bicolor *[44], *Malus *x domestica (Troggio, unpublished), *Zea mays *[78], and *Homo sapiens *[79,80]. Whole genome shotgun reads of each species were retrieved from the NCBI Trace Archive and converted to 60-bp lengths. 0.036× genome equivalents of these "reads" were then aligned to 920000 bp (similar to the total *P. taeda *BAC sequence) of randomly-selected regions of each genome using BLAST. Alignments were categorized into three nucleotide identity groups: 70-84%, 85-97% and 98-100%. The genomic sampling was conducted 10 times and averaged.

To simultaneously visualize all elements that were identified in the BAC assemblies, the program gff2ps was used [81]. The following data were formatted into GFF files and used to create Figure 1 and Additional file 1, Figure S1: MAKER dicot and monocot runs on masked and unmasked sequence; simple repeats; tandem repeats; direct repeats (potential LTRs); nongenic ORF elements; and coverage maps of each BAC at 75% identity and 99% identity. The coverage maps are shown in these figures as histograms of average hits per base pair in 50-bp windows. The GFF files are available for interactive browsing or download at http://dendrome.ucdavis.edu/treegenes/gbrowse, where a modified version of the GMOD project GBrowse was implemented in the TreeGenes database to display the annotations [82,83].

## Authors' contributions

ASK conceived and designed analyses, performed analyses and drafted the manuscript. JLW assisted with bioinformatic analysis and implemented the web browser. GP made major contributions to the design of the study and to graphic representation of results. CH worked with MY to customize the MAKER annotation pipeline for this project. GEB performed the BAC library screening. CAL performed the promoter analysis on predicted genes. JH was responsible for the sequencing, assembly and finishing of BAC assemblies. CHL, along with DBN, conceived of the project and obtained funding to perform this study. IK performed all bioinformatic analyses of the whole genome shotgun sequences used in this study and assisted with drafting the manuscript. All authors read and approved the final manuscript.

## Supplementary Material

Additional file 1 **Figure S1**. Ten *P. taeda *BAC assemblies with gene predictions, repeat identification and WGS coverage profiles of the BACs, as described in the text. Click here for file

Additional file 2 **Table S1**. Ten genic amplicons used to probe the *P. taeda *BAC library for sequences similar to coding sequences. Click here for file

Additional file 3 **Table S2**. Summary of eighteen genes and gene fragments predicted by MAKER in ten *P. taeda *BAC sequences. Click here for file

Additional file 4 **Table S3**. Twelve of the peptides predicted by MAKER showed significant similarity to Interpro (E-value > 1e-05). Click here for file

Additional file 5 **Table S4**. Promoter analysis of twelve predicted genes that showed similarity to Interpro (E value > 1e-05). Click here for file

Additional file 6 **Table S5**. Consensus sequences of the three most common sequence elements in the *P. taeda *genome, assembled from WGS reads. Click here for file
