# Supplementary material for: The Pinus taeda genome is characterized by diverse and highly diverged repetitive sequences
Source: BMC Genomics. 2010 Jul 7;11:420. doi: 10.1186/1471-2164-11-420 (PMC2996948; doi:10.1186/1471-2164-11-420)
Supplement: Additional file 1 — Figure S1. Ten P. taeda BAC assemblies with gene predictions, repeat identification and WGS coverage profiles of the BACs, as described in the text. [file 1471-2164-11-420-S1.PDF]

Supplemental Figure 1. Ten P. taeda BAC assemblies with gene predictions, repeat identification and WGS coverage profiles of the BACs, as described in the text.

- Conifer-specific LTR repeats in black
- Angiosperm-derived gypsy-like elements in red
- Angiosperm-derived copia-like elements in green
- Unidentified LTR retroelements are boxed in gray
- Dotted lines suggest an unknown repetitive element.

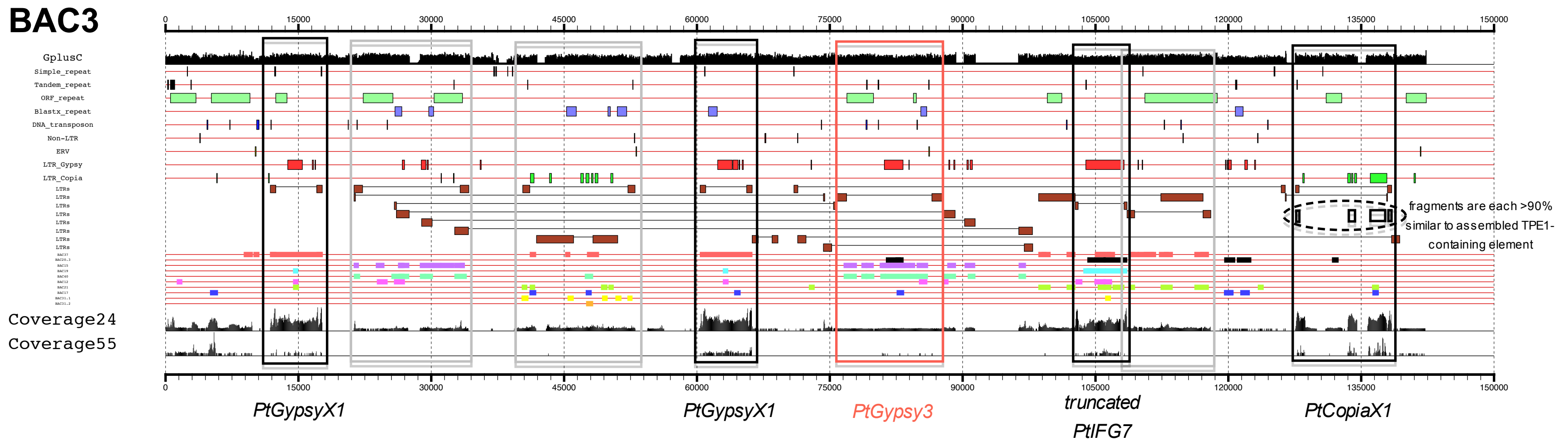
BAC3
0
15000
30000
45000
60000
75000
90000
105000
120000
135000
150000
GplusC
Simple_repeat
Tandem_repeat
ORF_repeat
Blastx_repeat
DNA_transposon
Non-LTR
ERV
LTR_Gypsy
LTR_Copia
LTRs
fragments are each >90% similar to assembled TPE1-containing element
Coverage24
Coverage55
PtGypsyX1
PtGypsyX1
PtGypsy3
truncated PtIFG7
PtCopiaX1

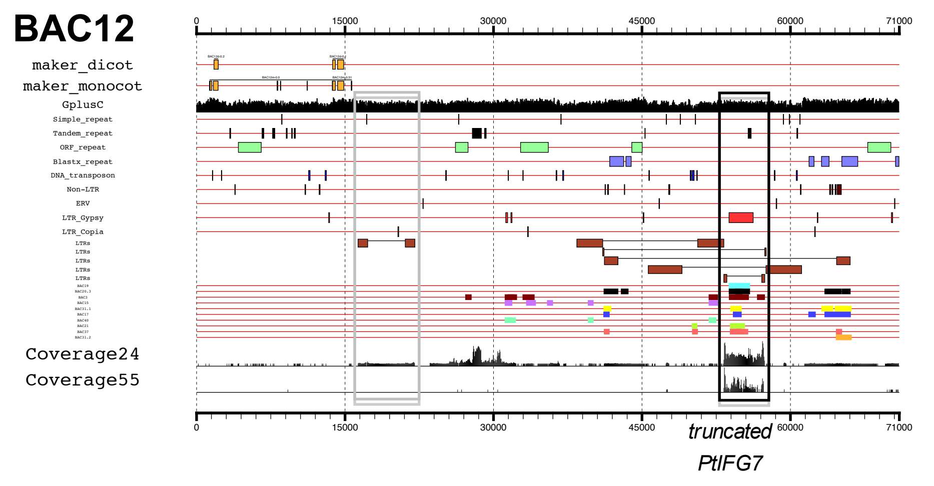

BAC12
maker_dicot
maker_monocot
GplusC
Simple_repeat
Tandem_repeat
ORF_repeat
Blastx_repeat
DNA_transposon
Non-LTR
ERV
LTR_Gypsy
LTR_Copia
LTRs
LTRs
LTRs
LTRs
LTRs
BAC19
BAC20.3
BAC3
BAC15
BAC31.1
BAC17
BAC40
BAC21
BAC37
BAC31.2
Coverage24
Coverage55
0
15000
30000
45000
60000
71000
truncated
PtIFG7

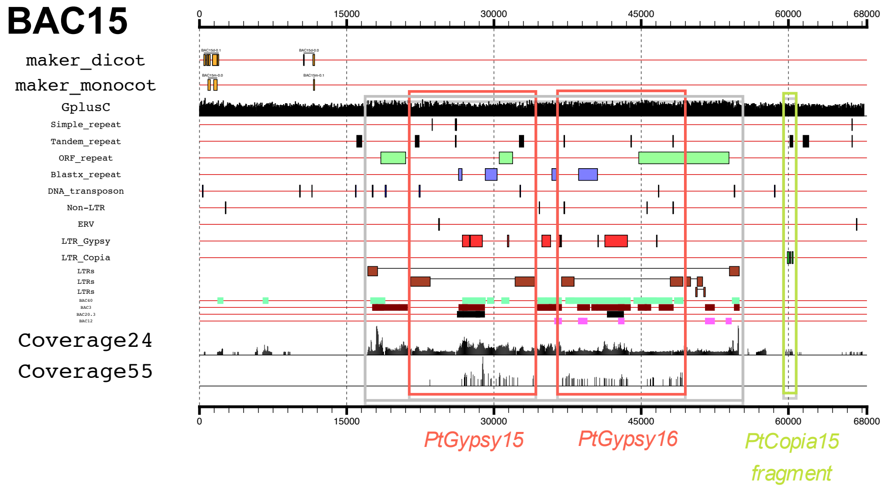
BAC15
maker_dicot
maker_monocot
GplusC
Simple_repeat
Tandem_repeat
ORF_repeat
Blastx_repeat
DNA_transposon
Non-LTR
ERV
LTR_Gypsy
LTR_Copia
LTRs
LTRs
LTRs
BAC40
BAC3
BAC20.3
BAC12
Coverage24
Coverage55
0
15000
30000
45000
60000
68000
PtGypsy15
PtGypsy16
PtCopia15
fragment

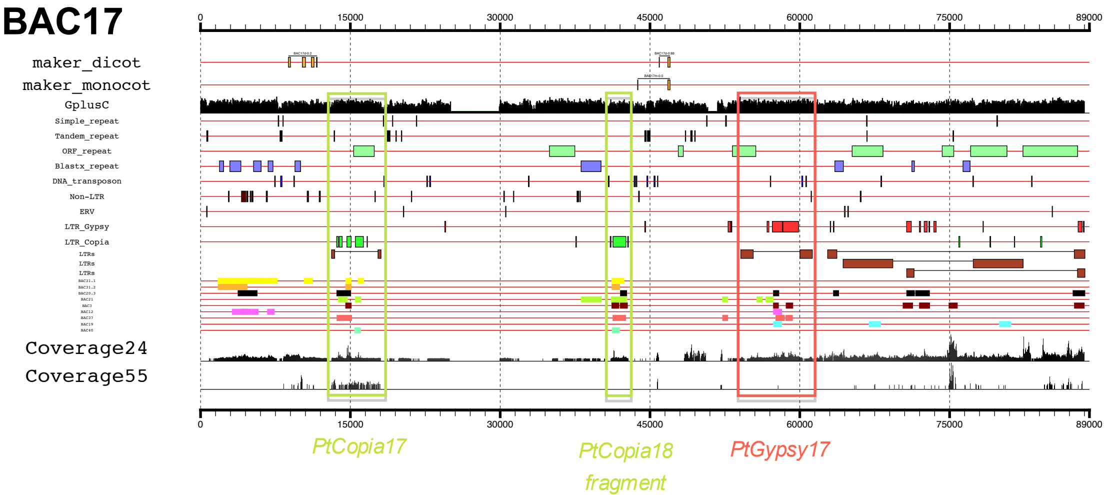
BAC17
maker_dicot
maker_monocot
GplusC
Simple_repeat
Tandem_repeat
ORF_repeat
Blastx_repeat
DNA_transposon
Non-LTR
ERV
LTR_Gypsy
LTR_Copia
LTRs
LTRs
LTRs
Coverage24
Coverage55
0
15000
30000
45000
60000
75000
89000
PtCopia17
PtCopia18 fragment
PtGypsy17

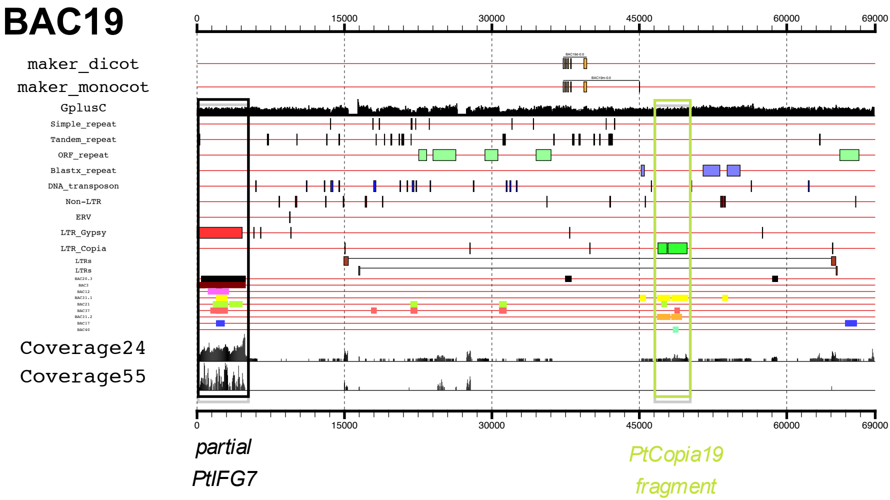

BAC19
0
15000
30000
45000
60000
69000
maker_dicot
maker_monocot
GplusC
Simple_repeat
Tandem_repeat
ORF_repeat
Blastx_repeat
DNA_transposon
Non-LTR
ERV
LTR_Gypsy
LTR_Copia
LTRs
LTRs
BAC20.3
BAC3
BAC12
BAC31.1
BAC21
BAC37
BAC31.2
BAC17
BAC40
Coverage24
Coverage55
partial
PtIFG7
PtCopia19
fragment

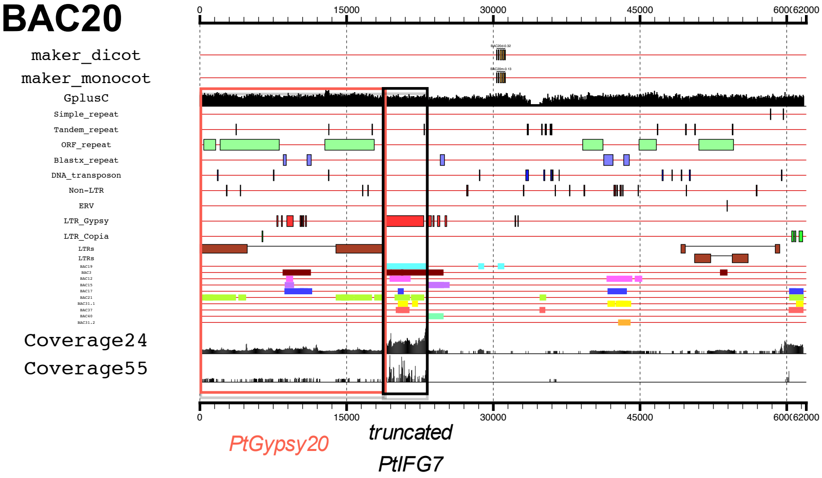

BAC20
maker_dicot
maker_monocot
GplusC
Simple_repeat
Tandem_repeat
ORF_repeat
Blastx_repeat
DNA_transposon
Non-LTR
ERV
LTR_Gypsy
LTR_Copia
LTRs
LTRs
BAC19
BAC3
BAC12
BAC15
BAC17
BAC21
BAC31.1
BAC37
BAC40
BAC31.2
Coverage24
Coverage55
0
15000
30000
45000
600(62000
PtGypsy20
truncated
PtIFG7

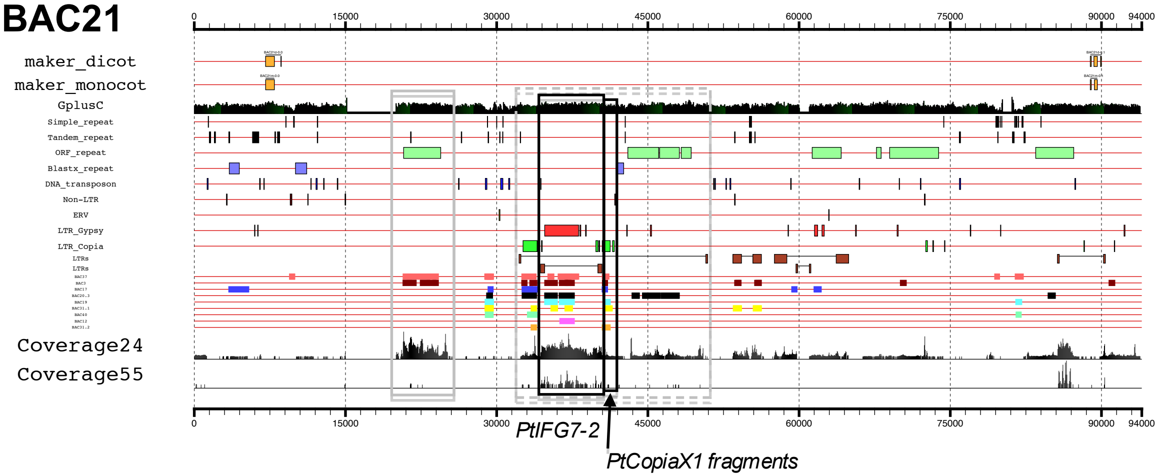
BAC21
0
15000
30000
45000
60000
75000
90000
94000
maker_dicot
maker_monocot
GplusC
Simple_repeat
Tandem_repeat
ORF_repeat
Blastx_repeat
DNA_transposon
Non-LTR
ERV
LTR_Gypsy
LTR_Copia
LTRs
LTRs
BAC37
BAC3
BAC17
BAC20.3
BAC19
BAC31.1
BAC40
BAC12
BAC31.2
Coverage24
Coverage55
PtIFG7-2
PtCopiaX1 fragments

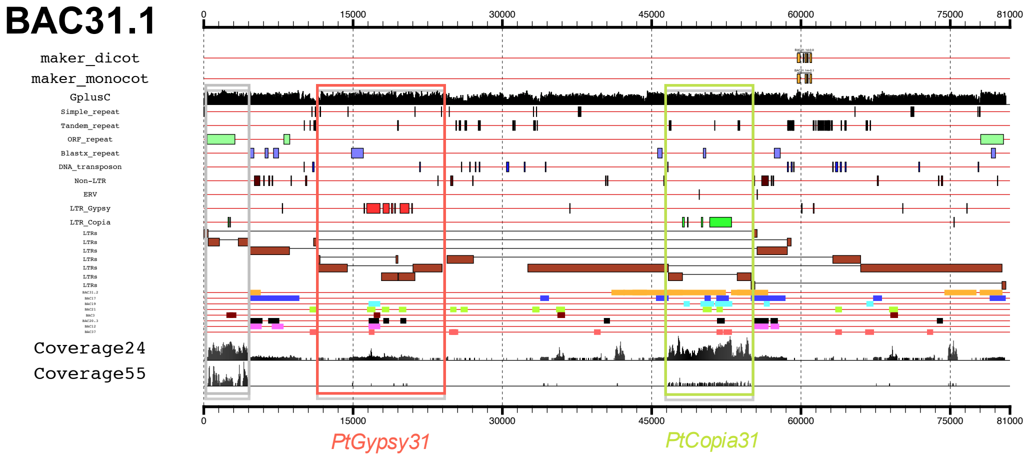

BAC31.1
0
15000
30000
45000
60000
75000
81000
maker_dicot
maker_monocot
GplusC
Simple_repeat
Tandem_repeat
ORF_repeat
Blastx_repeat
DNA_transposon
Non-LTR
ERV
LTR_Gypsy
LTR_Copia
LTRs
LTRs
LTRs
LTRs
LTRs
LTRs
LTRs
BAC31.2
BAC17
BAC19
BAC21
BAC3
BAC20.3
BAC12
BAC37
Coverage24
Coverage55
PtGypsy31
PtCopia31

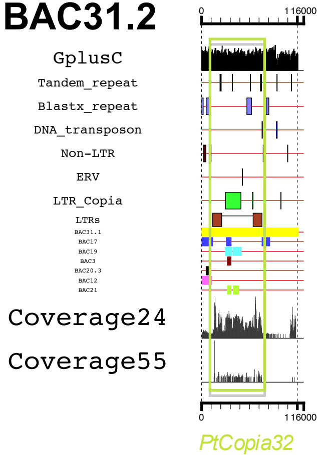
BAC31.2
0
116000
GplusC
Tandem_repeat
Blastx_repeat
DNA_transposon
Non-LTR
ERV
LTR_Copia
LTRs
BAC31.1
BAC17
BAC19
BAC3
BAC20.3
BAC12
BAC21
Coverage24
Coverage55
0
116000
PtCopia32

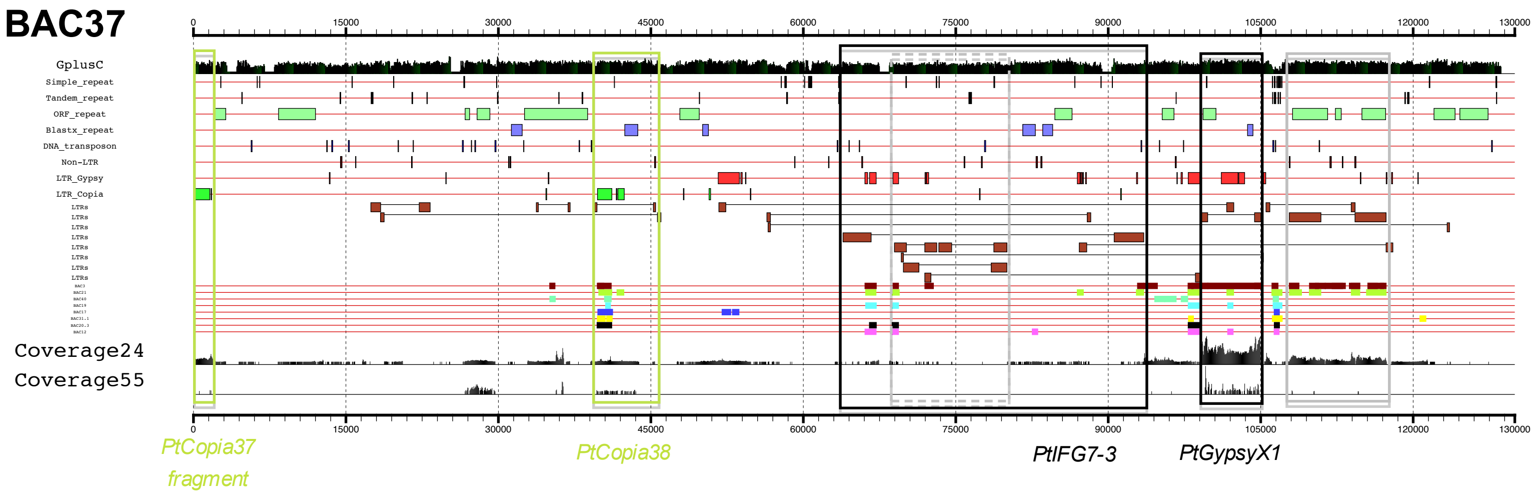
BAC37
GplusC
Simple_repeat
Tandem_repeat
ORF_repeat
Blastx_repeat
DNA_transposon
Non-LTR
LTR_Gypsy
LTR_Copia
LTRs
Coverage24
Coverage55
0
15000
30000
45000
60000
75000
90000
105000
120000
130000
PtCopia37 fragment
PtCopia38
PtIFG7-3
PtGypsyX1

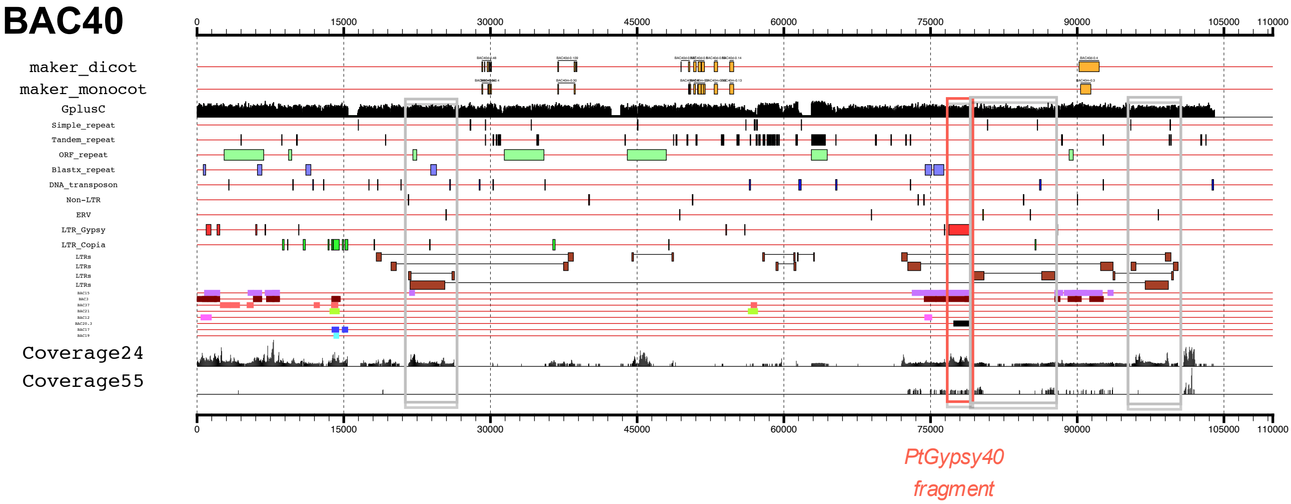
BAC40
0
15000
30000
45000
60000
75000
90000
105000
110000
maker_dicot
maker_monocot
GplusC
Simple_repeat
Tandem_repeat
ORF_repeat
Blastx_repeat
DNA_transposon
Non-LTR
ERV
LTR_Gypsy
LTR_Copia
LTRs
LTRs
LTRs
LTRs
BAC15
BAC3
BAC37
BAC21
BAC12
BAC20.3
BAC17
BAC19
Coverage24
Coverage55
PtGypsy40
fragment
