# Supplementary material for: The Pinus taeda genome is characterized by diverse and highly diverged repetitive sequences
Source: BMC Genomics. 2010 Jul 7;11:420. doi: 10.1186/1471-2164-11-420 (PMC2996948; doi:10.1186/1471-2164-11-420)
Supplement: Additional file 3 — Table S2. Summary of eighteen genes and gene fragments predicted by MAKER in ten P. taeda BAC sequences. [file 1471-2164-11-420-S3.DOC]

| **Dicot-like gene ID** | **Dicot genomic length (bp)** | **Dicot mRNA length (bp)** | **Dicot no. exons** | **Potential Gene Product** | | **Most Similar Protein (blastx)** | **Monocot no. exons** | **Monocot mRNA length (bp)** | **Monocot genomic length (bp)** | **Monocot-like gene ID** |
| --- | --- | --- | --- | --- | --- | --- | --- | --- | --- | --- |
|  |  |  |  |  |  |  |  |  |  |  |
| 12d0.2 | 1039 | 933^5UTR^ | 1 | 4-coumarate-–CoA ligase 1 | | Q42524|4CL1_ARATH | 7 | 909^5UTR^ | 12547 | 12m0.0 |
| 12d0.1 | 1115 | 888 | 2 | O-methyltransferase 2 | | O23760|COMT1_CLABR | 3 | 969 | 1944 | 12m0.31 |
| 15d0.0* | 1670 | 775^5UTR,3UTR^ | 2 | LIM transcription factor | | P29675|SF3_HELAN | 1 | 775^5UTR,3UTR^ | 1670 | 15m0.1 |
| 15d0.1 | 1517 | 1047 | 5 | Glycosyl transferase | | Q2TAA5|ALG11_HUMAN | 2 | 600^5UTR^ | 944 | 15m0.0* |
| 17d0.89 | 1089 | 234 | 2 | O-methyltransferase 2 | | Q6ZD89|OMT1_ORYSJ | 2 | 249 | 3248 | 17m0.0 |
| 17d0.2 | 2869 | 822 | 4 | Reverse transcriptase | | P41653|YCF2_PINTH | n/a |  |  |  |
| 19d0.0 | 2519 | 785^3UTR^ | 5 | O-methyltransferase 3 | | Q9ZTT5|CAMT_PINTA | 6 | 684^3UTR^ | 7775 | 19m0.0* |
| 20d0.32 | 1516 | 709^5UTR,3UTR^ | 5 | O-methyltransferase 3 | | Q9ZTT5|CAMT_PINTA | 5 | 709^5UTR,3UTR^ | 1516 | 20m0.13 |
| 21d0.1 | 1105 | 525 | 3 | Helicase SMARCA3 | | Q9FIY7|SM3L3_ARATH | 2 | 390^3UTR^ | 699 | 21m0.1* |
| 21d0.0 | 1547 | 897 | 2 | S-adenosylmethionine synthetase | | A2Y053|METK1_ORYSI | 1 | 873^5UTR,3UTR^ | 871 | 21m0.0* |
| 31d0.0* | 1453 | 764^3UTR^ | 5 | O-methyltransferase 3 | | Q9ZTT5|CAMT_PINTA | 4 | 764^5UTR^ | 1453 | 31m0.1* |
| 40d0.109 | 1962 | 282 | 3 | Unknown | | Q37680|NU5M_WHEAT | 2 | 204 | 1794 | 40m0.30 |
| 40d0.127 | 902 | 144 | 2 | Unknown | | Q9LYN8|EXS_ARATH | 3 | 201 | 221 | 40m0.0 |
| 40d0.1 | 1111 | 858 | 3 | LRR-containing protein kinase | | Q9LYN8|EXS_ARATH | 5 | 924 | 1178 | 40m0.1 |
| 40d0.13 | 348 | 348 | 1 | Protein tyrosine kinase | | Q9LYN8|EXS_ARATH | 1 | 348 | 348 | 40m0.12 |
| 40d0.14 | 414 | 414 | 1 | Protein kinase | | Q9LYN8|EXS_ARATH | 1 | 414 | 414 | 40m0.13 |
| 40d0.4 | 1065 | 2058 | 1 | Unknown | | Q06852|SLAP1_CLOTH | 1 | 1053 | 1053 | 40m0.3* |
| 40d0.48 | 985 | 369 | 5 | O-methyltransferase 2 | | P46484|COMT1_EUCGU | 2 | 291^5UTR,3UTR^ | 337 | 40m0.4* |
|  |  |  |  |  |  |  | 2 | 156^3UTR^ | 678 | 40m0.36 |
| **SUM** | **24212** | **12852** | **52** |  |  | | **50** | **9604** | **38690** | **SUM** |
| **AVE.** | **1346** | **714** | **2.9** |  |  |  | **2.8** | **565** | **2149** | **AVE.** |
| **ST DEV.** | **637** | **433** |  |  |  |  |  | **301** | **3116** | **ST DEV.** |

**Supplemental Table 2. Summary of eighteen potential genes identified by MAKER in ten *P. taeda* BAC sequence.** Also indicated are the Genbank identifiers of proteins that showed significant blastx similarity to each gene region (E-value > 1E-10). Gene designations given in the left- and rightmost columns indicate the BAC (first two digits), the parameters used in annotation (d/m) and a gene ID produced by MAKER (0.xxx). Transcripts that show > 97% nucleotide identity to *P. taeda* ESTs are highlighted in green.

^*^The gene structure predicted by MAKER lacks a consensus start codon (ATG) or a consensus stop codon, or both.

^5UTR^mRNA length in these putative genes includes between 1 and 1007 bp (in this set of genes) of predicted 5’ UTR sequence.

^3UTR^mRNA length includes between 1 and 549 bp of predicted 3’ UTR sequence.
