# Supplementary material for: The Pinus taeda genome is characterized by diverse and highly diverged repetitive sequences
Source: BMC Genomics. 2010 Jul 7;11:420. doi: 10.1186/1471-2164-11-420 (PMC2996948; doi:10.1186/1471-2164-11-420)
Supplement: Additional file 4 — Table S3. Twelve of the peptides predicted by MAKER showed significant similarity to Interpro (E-value > 1e-05). [file 1471-2164-11-420-S4.DOC]

| **Dicot-like gene ID** | **Dicot peptide length (aa)** | **Dicot Interpro E-value** | **Interpro signature** | **Interpro description** | **Monocot Interpro E-value** | **Monocot peptide length (aa)** | **Monocot-like gene ID** |
| --- | --- | --- | --- | --- | --- | --- | --- |
|  |  |  |  |  |  |  |  |
| 12d0.2 | 142 | 9.50E-16 | IPR000873 | AMP-dependent synthetase and ligase | 7.40E-13 | 275 | 12m0.0 |
| 12d0.1 | 295 | 2.00E-61 | IPR001077 | O-methyltransferase, family 2 | 2.00E-61 | 322 | 12m0.31 |
|  |  | 5.30E-10 | IPR012967 | Plant methyltransferase dimerisation | 5.30E-10 |  |  |
|  |  | 2.10E-19 | IPR011991 | Winged helix repressor DNA-binding | 2.10E-19 |  |  |
|  |  | ns | IPR016461 | O-methyltransferase, COMT, eukaryota | 4.80E-78 |  |  |
| 15d0.0* | 74 | 5.30E-05 | IPR001781 | Zinc finger, LIM-type | ns | 37 | 15m0.1 |
| 15d0.1 | 348 | ns | IPR001296 | Glycosyl transferase, group 1 | 2.30E-05 | 199 | 15m0.0* |
| 17d0.89 | 77 | 4.70E-08 | IPR001077 | O-methyltransferase, family 2 | 1.10E-08 | 82 | 17m0.0 |
| 19d0.0 | 222 | 3.10E-105 | IPR002935 | O-methyltransferase, family 3 | 3.70E-104 | 228 | 19m0.0* |
| 20d0.32 | 152 | 5.20E-79 | IPR002935 | O-methyltransferase, family 3 | 5.20E-79 | 152 | 20m0.13 |
| 21d0.0 | 298 | 3.40E-92 | IPR002133 | S-adenosylmethionine synthetase | 3.40E-92 | 290 | 21m0.0* |
|  |  | 3.30E-74 | IPR002133 | S-adenosylmethionine synthetase | 3.30E-74 |  |  |
| 40d0.1 | 285 | 5.00E-11 | IPR013210 | Leucine-rich repeat, N-terminal | 7.30E-07 | 307 | 40m0.1 |
| 40d0.13 | 115 | 1.70E-13 | IPR011009 | Protein kinase-like | 1.70E-13 | 115 | 40m0.12 |
|  |  | 1.80E-06 | IPR001245 | Tyrosine protein kinase | 1.80E-06 |  |  |
| 40d0.14 | 137 | 1.20E-17 | IPR011009 | Protein kinase-like | 1.20E-17 | 137 | 40m0.13 |
| 40d0.48 | 122 | 2.00E-10 | IPR001077 | O-methyltransferase, family 2 | 5.90E-10 | 81 | 40m0.4* |

**Supplemental Table 3. Twelve of the peptides predicted by MAKER showed significant similarity to Interpro protein signatures (E-value > 1E-05).** The remaining nine predicted peptides failed to show significant similarity to Interpro and are not shown here. Transcripts that were > 97% identical to *P. taeda* ESTs are highlighted in green.

*The gene structure predicted by MAKER lacks a consensus start codon (ATG) or a consensus stop codon, or both.
^5UTR^mRNA length in these putative genes includes between 1 and 1007 bp (in this set of genes) of predicted 5’ UTR sequence.

^3UTR^mRNA length includes between 1 and 549 bp of predicted 3’ UTR sequence.
