# Supplementary material for: The Pinus taeda genome is characterized by diverse and highly diverged repetitive sequences
Source: BMC Genomics. 2010 Jul 7;11:420. doi: 10.1186/1471-2164-11-420 (PMC2996948; doi:10.1186/1471-2164-11-420)
Supplement: Additional file 5 — Table S4. Promoter analysis of twelve predicted genes that showed similarity to Interpro (E value > 1e-05). [file 1471-2164-11-420-S5.DOC]

**Supplemental Table 4. Summary of promoter analysis of sequence immediately upstream of twelve genes annotated with dicot parameters and showing similarity to Interpro signatures (E value > 1e-10).** Similarity within promoters to EST or cDNA sequences can indicate that the predicted gene was annotated incorrectly or that it is a pseudogene. The presence or absence of TATA and CAAT boxes can signify either incorrectly annotated genes or inactive pseudogenes. Transcripts that showed > 97% nucleotide identity to *P. taeda* ESTs are highlighted in green.

|  | Promoter similarity (BLAST) | TATA? | CAAT? | PLACE elements |
| --- | --- | --- | --- | --- |
| 12d0.2/12m0.0-4cl1 | none | no | no | disease and abiotic stress elements |
| 12d0.1/12m0.31-comt | 3’ end sim. to comt cDNAs | no | yes | potential disease response elements, some drought response elements |
| 15d0.0/15m0.1-LIM_tf | intron in 5’ UTR? | yes | no | hormone response elements, MYB binding sites, myc binding sites |
| 15d0.1/15m0.0-gl_transf | none | yes | yes | drought stress and disease response elements |
| 17d0.89/17m0.0-comt | many P. taeda ESTs | yes | yes | gibberellin, cytokinin and ethylene response elements |
| 19d0.0/19m0.0-ccoaomt | 5’ end sim. to cDNAs | no? | no? | quite a few myb and my recognition elements including a few TGCAGG (involved in the phenypropanoid pathway) |
| 20d0.32/20m0.13-ccoaomt | 5’ end sim. to cDNAs | yes | yes | myb-binding element in the PAL promoter, myb binding site in promoters of several phenylpropanoid genes, other myb binding sites, myc binding sites including drought response, several auxin response, cytokinin responsive, ABA/drought responsive, elicitor response, ethylene response, gibberellin response, many W box elements (disease), and others |
| 21d0.0/21m0.0-sams | 3’ end of promoter to EST | yes | yes | myb, myc elements |
| 40d0.1/40m0.1-LRR_kin | none | yes | yes | disease response and elicitor response elements |
| 40d0.13/40m0.12-tyr_kin | none | no | yes | ethylene response, defense, sugar repression, WRKY-binding (usually defense), light response and xylem formation elements |
| 40d0.14/40m0.13-kinase | EST seen twice in promoter | no | no | Quite a few interesting elements. |
| 40d0.48/40m0.4-comt | 5’ end sim. to many ESTs | no | no | none, only cDNA sequence |
