# Supplementary material for: The Pinus taeda genome is characterized by diverse and highly diverged repetitive sequences
Source: BMC Genomics. 2010 Jul 7;11:420. doi: 10.1186/1471-2164-11-420 (PMC2996948; doi:10.1186/1471-2164-11-420)
Supplement: Additional file 6 — Table S5. Consensus sequences of the three most common sequence elements in the P. taeda genome, assembled from WGS reads. [file 1471-2164-11-420-S6.DOC]

Supplemental Table 5. Consensus sequences assembled from whole genome shotgun sequences.

| >*Pinustaeda*_*TPE1*copia-like_WGS_assembled_consensus  CCACTTGTAGATCTTCTCTGATCCAGATCTCCAGCCCAGTCTGCATCAACAAAGCCACGAATATCCAACACTCTGTCCAATCCTGGTCTTCCTTGGTAGCACAAACCATAATCACTAGTGCCACGCAAATACCTGAAAACCCGCTTCACTGTTGTCCAGTGCTCCTTCCCTGGTTTTGACATAAACCTGCTCAATACTCCCACTGCATGTGCAATGTCTGGTCTAGTACAGACCATTGCATACATCAAGCTACCGACTGCACTTGCATATGGAACACGGGACATGTCCTCTTCCTCTTCTTGTGTCTTGGGACACTGTTCAGCAGATAACCTCACACCTACAGGAATAGGAACCTTCACCGGTTTACTATCCTGCATATTAAACCTCTGCAGTATTGTCTCGACATACTTCCTCTGGTTTAACCAGAGCTTCCTCTTTGCCCGATCTCTTTTAATTTCCATGCCCAAAATATAGTTTGCAGCACCGAGATCTTTCATGTCGAATTTGGAGGACAATTGGGTCTTCAAATCCTGGATAATTTCTTTGTCATTTCCAACCAGCAACATATCATCCACATACAAGACCAGATAAATAACACGGTCACCAATTAATTTAAAATACACACAATGATCTGCTTTGCTTCTGGTGAAGCCAAGTCCCCGTATAAATGTATCAAATTTCTGATACCACATCCTTGGTGACTGTTTTAACCCATACAGAGACTTCTTTAACTTGCATACCAGTTCTTTCTTACCCTTCACCGCAAAGCCTTCAGGTTGCTTCATGTAGATTTCCTCTTCCAAATCCCCGTGAAGAAATGTTGTCTTCACATCCATCTGTTCTACCTCAAAATCAAAAGCAGCAGCAACAGATAAAAGTAATCTAATAGAAGTTACCTTGGCAACAGGAGAAAAAATATCACCAAAATCAATTCCCGGCACCTGGGAATAACCTTTTGCTACCAACCGAGCTTTGTATTTCTCCACCTTCCCTTCTGCATTTGTCTTCTTCTTGAACACCCATTTGCTGCCAATGGGTTTCCTTCCAGCCGGCAACTCCACCAGATCCCAAGCCTCATTTTTGTGCAAAGATGCCATTTCATCAACCATGGCCTCTTTCCAGAGTTTTCCATCCTCTGAATCCACTGCTTCCTTCACAGTTCTGGGATCATCATCAGTAACAGACAAAGCAAAATTTGAACAGAAAGCAGATGGACTATACCTTTCTGGCTGCCTTCTTTCTCGAACTGATCTTCTCACAGCCGGAGTTTGTGGCTCTTCATCTTCTGATTCCTCCTCTGCTACAGAGTCTGATTCTTCCTCCTTTAGTTCAAACTCTATCTTCACAGGTTCCTTTGGTTGGACTTCATGTTTAATAACATCTTTCACCTCTCTGAACACCACATCTCGACTGTACACAACCTTCCTTGTTACTGGGTTCCAAAGCTTATAACCCTTTAAACCATCCTTATACCCAATAAAGATGCACTTTTCAGATTTACTATCCAACTTGGTTCGTTTCTCCTTTGGAACATGTACATATGCATCACATCCAAATACCCTCAGATGTGAAAGAGAGGGTTTCTTACCAGTCCATACTTCTTGTGGAGTCTTATCCTCCAACGCTGATGAAGGTGACCTGTTGACCAAGTAACATGCAGTATCCACCGCCTCTGCCCAGAATTCCTGTCCTAACCCGGCACCACTGAGCATGCTTCTTGCCCTTTCCATCAACGTCTTGTTCATTCTTTCTGCGACTCCATTTTGCTGAGGTGTATATGGAGTAGTCTTCTGCCGTGCTATACCACACTTCTTACAGAATTCTTCAAATTCCTTGCTGCAGAATTCTCCACCATTATCTGTCCTCAGCACCTTTATCTTCTTCTCTGTCTGATTTTCCACCAGAGCCTTAAATTCTTTAAACCTGTCAAAGACTTCAGATTTCTTCTTAAGGAAATATATCCATGTATTCCTTGAGAAGTCATCTATAAATGATACATAGTACACAGACTTACCCAGTGATGGAACCTTCACAGGTCCAAACACATCACTGTGCACAAGCTCTAATATTTGTTTCGTCCTCTTACCACCAGAGGGGAAACTCACCCGATTCTGCTTCCCATATACACAATTTTCACAGAAATCAAAATCCAGAGAGCTATTACTCATACCTTCTACCATACCTTTACCATGAAGTATTCGAAGGCCCTTCTCTCCAATATGTCCAAGCCTTTGATGCCACAACATGGTCTTCTCTCCAGAGACCACTAGATTTTCTGCTCCATTTTCAGGAACCATAGAACTATTGCACCCGTCAACAACAGTGCTACCTTGCAGCTTGTACAGAGTTCCAATCCGAACTCCCCGCATCAATACCAGTGCTCCTCGAACCATCTTGCAGGTATCCTTCTCGAACACTGTCTTTACACCTGCATCATCCAACTTGCTTACAGAAATCAGATTTCTGGCCAATGCAGGAATATGCAGAACACCCGGAAGGGTTCTAACCCTTCCACCTTGCAACTTCAACTTTACTTTTCCACGACCAATGATTCTAGCCTTCCTATCATCTCCTAAGAAGACATCACCACCATCATATTTCTCATACTCGCAGAACCACTCTCTATGGGGAGTGAAGTGAAAGGATGCACCTGAGTCAATCAACCATGCCTCATGATCTACATGTGTACTTGAAGAAGCCAAGTACACATCCCCACCTTCATCTGAGGTGGTTTTCGCCTCTGCAGAAGGAGCATCATCAGATCCCTTTCCTTTATCAGGAGCTTTAGATTTACACTCCCTTCTGAAGTGCCCTTCCTTTCCGCATTTCCAGCACACCACCTTTATAGGTTTTCCTGGAGATTTAGATCTCCCTCTAGATTTAGATCTCCCACTCGATGACTTATTTTTGTTTCTGTTCTGGGACCGTCCTCGTACAGACAAGGCATCACCATTCTGGTTGTCCATATTTTTCCGTCTCATCTCCTCCGTCAGCAAGGCTGAGACTATTTCATCAAACTGCAAAGCAGTTGCATTGCTACCTATTGCAATAACCAGACTATCCCACGAATCTGGTAGAGAACACAACAAACTGATGCACTTATCTTCATCTGAAATTTTAATATCAACAGATGATAACTGACTTACCACAGTATTAAACGCATTCAGATGCTCTGTCACCGAGTCTCCATCTTTCATCCTCAGGTTATACAGCTTTTTCCGCAGGAACAGTTTATTCACCAGAGACTTAGATTGGTACAAAGTCCCTAACTTGTCCCACAAAGCCTTCGCCGTAGCTTCCCCTGATACATTCAACAATACTGAATCTGAGACACACAATCGTATTGTGCTCTTTGCCTTCCGATCCAACTTCTTCCATTCTTCATCGGTCACTCCCGTTGGTTTAGTACCCGGATCTACCGCGATCCACTGATCTTTATCCACCAACAGATCCTCCATCTTGAGTTTCCACAACTCAAAACTCTGACCATTGAACTTCTCAATCTCTATCTTTCCGCTGCTCGCCATCTTCTTTCACCACCAGAATCGATCTCGCAGATCTCCCACTGAACAGGGTCTACCACAGGCCCGAGCGAAACCCCTCAGCAACAAAGGCGTCCCCTTGAAGCCAAAACAGCTCTGATACCAAATGTAGGTTTTAACCCCGTACAGAAATTACCTGCATACGAATTCTAAATCACAGGAAAAATAAAAATGCGAGAGCATAACACATAAAACACATATACACAGAGATTTCCCACTGGATCTCAAAACCGAGACTACCAGCTTGATTTTTCATTATGTTCCTTTCAATGTTACAATACATATTTATACATGCCGAAGCATCCAATACCATGGACCGAAAACCATCGCGTCCAAAACCATGGACCGAAAACC |
| --- |
| >*PtIFG7*_WGS_assembled_consensus  CTAGATAAAGAAGAAATGAATCCGACCATATCTTGTAATGCATTGGCGGGAATTACCACTCCTCAAACCATCAAGATAGAAGGACAAATCAAGAAGAAAAAGGTAATAGTGTTGATTGATTCAGGAAGTACCCACAATTTTATTCATTGTAAGGTAGCAAAAGAATTGAATTGCTTCCTATATCCAGCACCAGAGTGTCAAGTGATGGTTGCAAATGGAGGAACAATAAATTGCTCTGGAAAGTGCCATAATATCAAGCTATCCATGGGAGAATATGTATTGACTAGCCCAATGCTTTCCATTCCAATGGGAGGTGCTGATGTTGTACTAGGAGTCCAATGGCTACAATCCTTGGGTACAATAGCTTTTAATTTTCAAGAACTTTTCATGAAATTTTCTATGGAAGGAAAGGAAGTTGAATTAAGGGGTATTGCAGGGAAACCAGGAAAGATAATCAGCTCTAATGGTATGACAAAGCTTCTAAAAAAGGAACAAAGAGGTGTAATTGCACAATTATGTTCGCTAGATGTTTCCACATTGGAATCATCTATTTCTCCAGATCTCCAAAAAGTCTTGGACAATCATTCCAAGGTATTTGAGACTCCCAAAGGTCTCCCACCTATTCGTGATCATGATCATGCTATTCATCTGATTCCAGGAAGTGTTCCTCCAAACATCAGGCCGTACAGATATCCCTATGCCCAAAAGAGTGAAATTGAACGTATGGTTGCAGAAATGCTAGAGGCTGGTATAATTCAACCTAGTCAAAGTTCTTTCTCTGCTCCAGTAGTATTGGTGCACAAGAAGGATGGATCATGGCGCATGTGTCCGGATTATAGGGAGCTCAACAAGCTCACTATTAAAGATAAGTTTCCCATTCCTGTCATTGATGAATTACTGGATGAATTGCATGGATCAATTTACTTCACCAAGTTGGATCTTCGTTCAGGATATCATCAAATCAGAATGAAGACTGAAGACATTCCGAAAACAACATTTAGAACTCATGAGGGTCATTATGAATTTTTGGTCATGCCTTTTGGCCTTACCAATGCACCTTCAACATTTCAAGGTTTGATGAATTCAATTTTCAAACCATTCCTTAGAAAATTTGTGTTAGTATTTTTTGATGATATACTAATCTACAACAAGTCTTGGAAGGATCATGTTGAACATGTTGACAGGGTGCTACAACTATTGGAGGAGAAACAATTATATGCAAAAAGATCCAAATGTTTCTTTGGAGTACAAGAGGTGGAATATCTGGGTCATATTGTATCTCATGAAGGAGTTAAGGTAGACCCTAGCAAAATTAAAGCCATTAAGGAATGGAAAATCCCCACATCCATAAAGCATCTGCGAGGATTTCTCGGGTTGACAGGGTATTATCGCAAGTTTGTTAAGAATTATGGGAGAATAGCAGCACCTCTAACAACATTATTGAAGAAAGATGCATTTTCATGGACTCCAGAAGCAACAAAGGCCTTTGAACATCTTAAAGAGGCAATGTGCCAAGCACCGGTCTTAGCTACACCGGACTTCACAAAAACCTTTATTGTGGAATGTGATGCTTCAGGAAATGGAATTGGTGTTGTTTTAATGCAAGATGAAAGACCCATTGCTTTTGAAAGTCGTCCAATCAAGGGAAAGTTTTTACACAAAGCTATTTATGAGAAGGAAATGTTGGCAATACTCCATGCACTTAAGAAATGGCGACCCTACCTAATGGGAAGACACTTCAAGGTAAAAACGGATCATGATAGCCTTAAATACTTTTTAGAACAAAGATTATCCTCTGAAGAGCAACAAAAGTGGGTCACAAAGATGTTGGGTTATGACTTTGAAATCATCTACAAAAAAGGGAAGCAAAATGTGGTGGCAGATGCACTCTCAAGAAAGGATGAGGATGTGGAAGCATTGTTGTGTGCCATTTCTATTATCCAACCAGATTGGATAAACGAAGCAAGGGAGGAATGGAAGAATGACGAAGAAGTGTGGGCACTTATTCGAAAGTTACAACAAGATTCCAGTACGTCTGATACATTTAGCTGGAAAAATGATTCGTTATGGTACAAAGATCGCTTATACCTCTGTAAGAATTCCCAGCTAAAACAAAAGATTCTTATGGAATTGCACACTTCTCCCTTAGGAGGGCACTCAGGATTTTTAAAAACTTACCACAGGGTTAAGAAGGAATTTTTTTGGGATGGCCTTAAATCAGATATTCAAAAGTTTGTGGCGGAATGTTTGGTTTGCCAACAAAATAAAGTTGAAACAATTAAGACACCGGGTCTATTACAACCTTTATCCATTCCAAGCCAACGTTGGGAGGAGGTTTCAATGGATTTTATCACAGGTTTACCCAAGTCTGAGGGAAAGAGTGTCATCATGGTGGTAGTTGACAGACTTACCAAGTATGCACACTTTTGTGCATTATCACATCCATTTAAAGCCAGTACAGTTTCTACTGCATTTATGGAAACAATTCAAAAGCTACATGGAAACCCAAAGATTATTGTAAGTGACAGAGATCCCATTTTCACTGGAAATTTTTGGACAGAATTATTTTCTTGTCTTGGTACTCAGTTAGCTCATAGCTCATCTTATCATCCTCAATCTGATGGGCAAACTGAGATAGTAAACAAATGTTTGGAAGGATATCTTCGTTGCTTTGTATCTGATAAACAGACACAATGGGTCAAATGGTTGCCTCTGGCTGAATGGTGGTATAACACTTCCTTCCATACAGCAACAAAAATGACCCCATTTATGGCACTTTATGGATATCAGCCACCATCCATCACATCATATTTAAGAGAAAATTCTAAGGTTCAAGCAGTGGAACATCACATCGAACATCAACAACAAGTCCTCCAACTCTTAAAGGATAACCTCGTGTTGGCACAGAATAGAATGAAACAACAGGCAGATCAACATCGCAGTGAAAGAAGTTTTGATGTAGGTGATTGGGTATTCCTACGGCTACAACCATATAAGCAAATGTCCCTCAAGCAAGCTAAGAAGGATAATAAATTATCACCAAAGTATTATGGTCCGTACAAGGTGTTGCAAAAGATTGGTACTATGGCATACAAATTGGAGCTTCCTGCAGCTTCTCGATTGCACCCAGTTTTCCATGTTTCATGTTTAAAGAAGGTTATAGGTGACAAGCTCCCAGTTCAAACAATATTGCCAGAACTTGACGAGGAAGGAAAAATTATATTGGAACCGGAAGCAGTCACAGAAACAAGAACTCGACAGCTACGAAATCGATCAATTTCAGAGTATCTTATCAAGTGGAAGAACTTATCTGCTGAAGATTCCACATGGGAGGATGAGAATTTTATACAGAAGCATCCAGAACTACTCAAGCGTTGAGGACAACACTTTTTTGAAGGAGAGGGGCATGTTAGGTCCCTATAATACTAGTGATTACCCCATTACTATTGTAGTTGTACCCCTTATTAATATTGTTGTGCCCCTATTGTCCCCATATTGTTATTACTGTGCCCCATAGAATCCCTCTATTGTTATTTTTGTGGCTTTATATTATTATTGTTAGGTCGGCTGGGTTATATTGTGACTCCTTAGACTCCTATATATAGAAAGGAGGTTTTGTCATTTGTATCATCCAAGAATTGTTCATTTATGTAATGTAAA |
| >*Pinustaeda*_cen-rpt_WGS_assembled_consensus  AAAGATGACAAAAGATCATGAAAGATCATGAAAGATCATGAAAGATAATAGGAGGTTGCCCCCTCGAAGAGGCTGA |
